# Direct activation of the proton channel by albumin leads to human sperm capacitation and sustained release of inflammatory mediators by neutrophils

**DOI:** 10.1038/s41467-021-24145-1

**Published:** 2021-06-22

**Authors:** Ruiming Zhao, Hui Dai, Rodolfo J. Arias, Gerardo A. De Blas, Gerardo Orta, Martín A. Pavarotti, Rong Shen, Eduardo Perozo, Luis S. Mayorga, Alberto Darszon, Steve A. N. Goldstein

**Affiliations:** 1 Departments of Pediatrics and Physiology & Biophysics, Susan and Henry Samueli College of Health Sciences, University of California, Irvine, CA USA; 2 Instituto de Histología y Embriología de Mendoza (IHEM/CONICET-UNCuyo), School of Medicine, National University of Cuyo, Mendoza, CP Argentina; 3 Laboratorio de Telediagnóstico e Investigación Traslacional (LaTIT). Área de Farmacología. Departamento de Patología, School of Medicine, National University of Cuyo, Mendoza, CP Argentina; 4 Departamento de Genética del Desarrollo y Fisiología Molecular, Instituto de Biotecnología, Universidad Nacional Autónoma de México, Morelos, México; 5 Department of Biochemistry and Molecular Biology, Gordon Center for Integrative Science, University of Chicago, Chicago, IL USA

**Keywords:** Innate immunity, Reproductive biology, Molecular medicine

## Abstract

Human voltage-gated proton channels (hHv1) extrude protons from cells to compensate for charge and osmotic imbalances due metabolism, normalizing intracellular pH and regulating protein function. Human albumin (Alb), present at various levels throughout the body, regulates oncotic pressure and transports ligands. Here, we report Alb is required to activate hHv1 in sperm and neutrophils. Dose-response studies reveal the concentration of Alb in semen is too low to activate hHv1 in sperm whereas the higher level in uterine fluid yields proton efflux, allowing capacitation, the acrosomal reaction, and oocyte fertilization. Likewise, Alb activation of hHv1 in neutrophils is required to sustain production and release of reactive oxygen species during the immune respiratory burst. One Alb binds to both voltage sensor domains (VSDs) in hHv1, enhancing open probability and increasing proton current. A computational model of the Alb-hHv1 complex, validated by experiments, identifies two sites in Alb domain II that interact with the VSDs, suggesting an electrostatic gating modification mechanism favoring the active “up” sensor conformation. This report shows how sperm are triggered to fertilize, resolving how hHv1 opens at negative membrane potentials in sperm, and describes a role for Alb in physiology that will operate in the many tissues expressing hHv1.

## Abstract (web summary)

The human voltage-gated proton channel (hHv1) maintains intracellular pH and membrane potential in sperm and neutrophils. Here, the authors show that albumin activates hHv1, by binding to the channel voltage sensor domains to enhance open probability and increases proton current, and that activation is required to trigger sperm to allow oocyte fertilization and to sustain production and release of immune inflammatory mediators during the neutrophil respiratory burst.

## Introduction

Regulation of intracellular pH (pH_i_) is essential to cell biology in health and disease^1^ and, since its identification^2,3^, the human voltage-gated proton channels (hHv1) has been recognized to be both widespread and central in many of these processes^4^. Recently, we used a designed peptide inhibitor (C6) of the channel to demonstrate that H^+^ efflux via hHv1 is required in human sperm to induce intracellular alkalization and initiate capacitation, and essential in human neutrophils to maintain cytoplasmic pH during the respiratory burst to allow reactive oxygen species (ROS) production^5^, in support of earlier proposals^6–10^.

hHv1 channels are comprised of two identical subunits, each with four transmembrane spans (TMs) that resemble the voltage sensor domains (VSDs) in other voltage-gated ion channels (VGICs) but lack the two additional TMs that contribute to forming the ion conduction pores in those channels^2,3^. In hHv1, there are two H^+^-selective conduction pathways, one in each subunit^11–13^.

Albumin (Alb), is the most abundant protein in interstitial fluids where it is present at various levels (7–30 mg/mL)^14^ and human plasma (34–54 mg/mL) where it is recognized to transport hormones, metabolites and drugs, serves as a circulating antioxidant, and supports oncotic pressure^15^. This ubiquitous globular protein is composed of 585 amino acids and folds into three domains^16^.

We wondered about a physiological connection between Alb and hHv1 based on its contrasting concentrations in semen (just 1 mg/mL, 15 µM)^17^ and in uterine fluid (34 mg/mL, 500 µM)^18^ and the documented increase in reproductive success when in vitro fertilization (IVF) solutions are supplemented with Alb^19,20^. Furthermore, Alb is implicated in deleterious systemic inflammatory responses mediated by neutrophils after cardiopulmonary bypass and in periodontal disease^21,22^.

Here, we demonstrate that Alb binds directly to hHv1 to activate the channel, increasing the open probability and H^+^ current. In human sperm, this initiates capacitation while in human neutrophils it increases peak levels of ROS release, sustains ROS production during the respiratory burst, and stimulates release of proteases in response to immune stimulation. The stoichiometry of binding, inferred by the Hill coefficient for changes in H^+^ currents in response to Alb dose, is confirmed to be one Alb per channel using single molecule total internal reflection fluorescent (smTIRF) microscopy. Alb is shown to bind to hHv1 on the external residues linking the third and fourth transmembrane segments that comprise the voltage sensor (S3-S4 loop) in each subunit using point mutations and chimeric channels generated between hHv1 and the proton channel from *Ciona intestinalis*, CiHv1^3^. Modeling with molecular dynamics (MD) simulation points to two binding sites formed by residues in Alb domain II (DII) for the two S3-S4 loops in each hHv1 channel. The two Alb sites are validated by the effects of mutagenesis on binding as assessed by fluorescence resonance energy transfer (FRET) microscopy and electrophysiology. The structural model of the Alb-hHv1 macromolecular complex suggests an electrostatic gating modification mechanism through which binding of Alb facilitates opening of hHv1 channels by favoring the active “up” conformation of the two VSDs. The essential stimulatory role of Alb in the physiology of both sperm and neutrophils via hHv1 suggests that Alb will have as-yet unrecognized roles in the many other tissues where the channel is critical, including, the heart, the central nervous system, and cancers of the breast and gastrointestinal tract^23,24^.

## Results

### Alb acts on hHv1 in human sperm to initiate capacitation

Human sperm undergo a process called capacitation in the female reproductive tract^25^, whereby pH_i_ rises stimulating Ca^2+^ influx and mobility changes^26^. This process endows sperm with the capacity to undergo the exocytotic acrosomal reaction required to penetrate the zona pellucida and fertilize the oocyte^27^. Here, Alb was observed to activate hHv1 channels in live human sperm by recording proton currents in mature non-capacitated spermatozoa, using whole-cell patch clamp as previously described^5^.

**Fig. 1:**
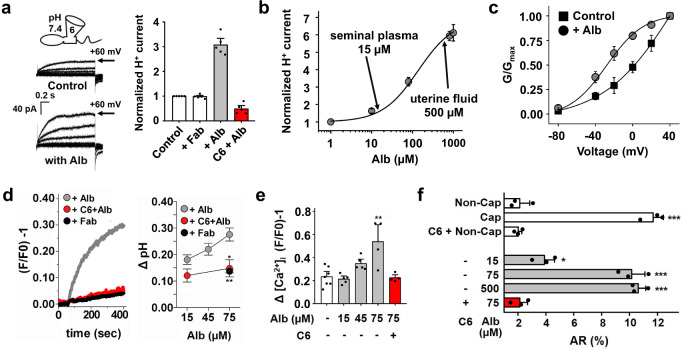
Alb activates hHv1 in human sperm to initiate capacitation and allow the acrosome reaction. **a** Left, Proton current traces in non-capacitated human sperm in the absence (top) and presence (bottom) of 80 μM Alb (20 mV steps from −60 and +60 mV). Right, hHv1 currents at the end of a test pulse to +60 mV with 80 μM Fab (white bar), 80 μM Alb (gray bar), or 80 μM Alb + 1 μM C6 (red bar). Values are normalized to mean current amplitude without Alb. C6 peptide blocked 86 ± 3% of the Alb-activated current, *n* = 5 cells. **b** Dose-response relationships for Alb potentiation of sperm proton current at +60 mV. The half-maximal effective concentration (EC_50_) of Alb activation estimated from the fit to Hill relationship as 158 ± 16 µM with a coefficient of 1.09 ± 0.01, *n* = 5 cells. **c** Conductance-voltage relationships (G-V) for sperm proton currents in the absence (black squares) or presence of 800 µM Alb (gray circles). Curves were fit to a Boltzmann equation, *n* = 4 cells. **d** Left, Non-capacitated sperm were loaded with BCECF and changes of fluorescence intensity measured. 75 μM Alb (gray trace) increased pH_i_ and the increase was inhibited by 20 μM C6 (red trace). A 75 μM Fab alone had no effect (black trace). Right, BCECF signals were converted to ΔpH as described in Methods. Alb-induced cytoplasmic alkalization was concentration dependent (gray circles). The Alb-triggered pH_i_ increase was inhibited by C6 (red circles, * *P* = 0.03; one way ANOVA, Dunnett Test); Fab did not increase the pH_i_ (black circle, ** *P* = 0.005; one way ANOVA, Dunnett Test), *n* = 3–13 independent experiments (Supplementary Fig. 1a). **e** Non-capacitated sperm were loaded with Fluo-3 and changes in fluorescence measured. Alb potentiates the increase of [Ca^2+^]_i_ induced by progesterone (15 μM) in a concentration dependent manner (gray bars). The [Ca^2+^]_i_ increase potentiated by 75 μM Alb was 2.3-fold (** *P* = 0.002; one way ANOVA, Dunnett Test) and was fully suppressed by 20 μM C6 peptide (red bar), *n* = 4–7 independent experiments (Supplementary Fig. 1b, c). **f** Acrosomal exocytosis induced by 15 μM progesterone. Upon progesterone stimulation control capacitated sperm underwent the acrosome reaction (Cap, white bar, *** *P* = 0.0001; one way ANOVA, Dunnett Test), whereas non-capacitated sperm did not (Non-Cap, white bar). Incubation of C6 peptide (20 μM) with non-capacitated sperm had no effect (C6 + Non-Cap, white bar). In a concentration dependent manner, incubation with Alb increased the progesterone-induced acrosome reaction (gray bars, * *P* = 0.04; one way ANOVA, Dunnett Test) in non-capacitated sperm; addition of C6 (20 μM, red bar) fully inhibited exocytosis stimulated by 75 μM Alb, *n* = 3 independent experiments. Values are mean ± SEM. Source data are provided in the Source Data file.

The native proton channel currents were elicited by depolarizing voltage steps of 1.5 s from a holding potential of −60 mV every 10 s with a ~30-fold proton gradient (pH_i_ = 6.0 and pH_o_ = 7.4). When 80 μM Alb was applied to the sperm, the currents increased ~3-fold, accompanied by a ~3-fold acceleration of activation and a ~1.6-fold slowing of deactivation at +60 mV (Fig. 1a, left; Supplementary table 1). Applying different concentrations of Alb yielded an EC_50_ = 158 ± 16 µM with a Hill coefficient of 1.09 ± 0.01. The mean concentration of Alb (15 µM) in human semen was too low to significantly activate hHv1, while the level in the uterus (500 µM) increased sperm proton current ~6-fold at +60 mV (Fig. 1b), as did 800 µM Alb in association with a half-maximal shift in the conductance-voltage (G-V) relationship (V_1/2_) of −32 mV (Fig. 1b, c). In contrast, addition of a control soluble protein of similar mass, the Fab fragment of human immunoglobulin G (Fab), had no effect on the proton currents in sperm, whereas Alb-activated currents were suppressed by 1 µM C6, indicating that they were passed by hHv1 channels (Fig. 1a, right). As H^+^ currents depend on driving force, and a variety of proton concentrations (pH_i_ and pH_o_) and cell types are studied in this report, the effects of Alb are collated in Supplementary table 1.

We next focused on the effect of Alb on pH_i_, as capacitation first requires H^+^ efflux via hHv1 to alkalinize sperm^5–7^. The fluorescent ratiometric pH probe BCECF was used to evaluate changes in the cytosolic pH, as previously described^28^. As anticipated, 75 μM Alb induced a robust increase in pH_i_ in non-capacitated human sperm when compared to a control Fab protein or after blocking hHv1 with C6 (Fig. 1d). Notably, increasing levels of Alb led to greater cytoplasmic alkalization consistent with a dose-dependent increase in H^+^ efflux and changes in pH_i_ were suppressed by the hHv1 channel blocker C6 (Fig. 1d, Supplementary Fig. 1a).

To confirm that Alb-induced changes in pH_i_ increased Ca^2+^ influx through CatSper^5–7^, non-capacitated sperm incubated with Alb were exposed to progesterone, a stimulus that triggers the acrosome reaction only if sperm have been capacitated by changes in pH_i_ and intracellular calcium levels ([Ca^2+^]_i_)^29,30^. In a concentration dependent manner, Alb enhanced progesterone-induced increases in [Ca^2+^]_i_, a change that was suppressed by the hHv1 blocker C6 (Fig. 1e, Supplementary Fig. 1b, c). Confirming that Ca^2+^ influx was mediated by CatSper, the increase in [Ca^2+^]_i_ was also inhibited by 1 μM NNC (Supplementary Fig. 1c). Verifying that Alb did not alter dye loading to produce an artefactual change in measured [Ca^2+^]_I_, applying the Ca^2+^ ionophore ionomycin to samples after progesterone stimulation produced the same absolute rise in peak [Ca^2+^]_i_ in the presence and absence of 75 µM Alb (Supplementary Fig. 1,d, e, f). Furthermore, Alb was shown to have no direct effect on CatSper currents in human sperm (Supplementary Fig. 2), consistent with a report that CatSper was sensitive to bovine serum albumin (BSA) indirectly^31^.

The final step, the exocytotic acrosomal reaction, requires prior capacitation^30^ and was rarely observed (~2%) when we added progesterone to non-capacitated sperm in the absence of Alb (Fig. 1f). In contrast, when 75 µM Alb was added to non-capacitated sperm ~10% of the sperm underwent exocytosis, an increase that was eliminated when sperm were incubated with the hHv1 blocker C6 (Fig. 1f). Thus, activation of hHv1 by Alb is essential for sperm alkalization and CatSper potentiation (that is, capacitation) and exocytosis (that is, the acrosomal reaction)^30^.

### Alb stimulates hHv1 in neutrophils to augment ROS production and elastase release

Human neutrophils undergo a respiratory burst to produce ROS as a principal effector mechanism to kill bacteria^32^. During the respiratory burst, the NADPH oxidase 2 (NOX2) transfers electrons across the membrane, resulting in membrane depolarization and cytoplasmic acidification that suppresses ROS production^33^. To sustain NOX2 activity and ROS production, H^+^ efflux is required to maintain physiological pH_i_ and membrane potential^23,34^.

**Fig. 2:**
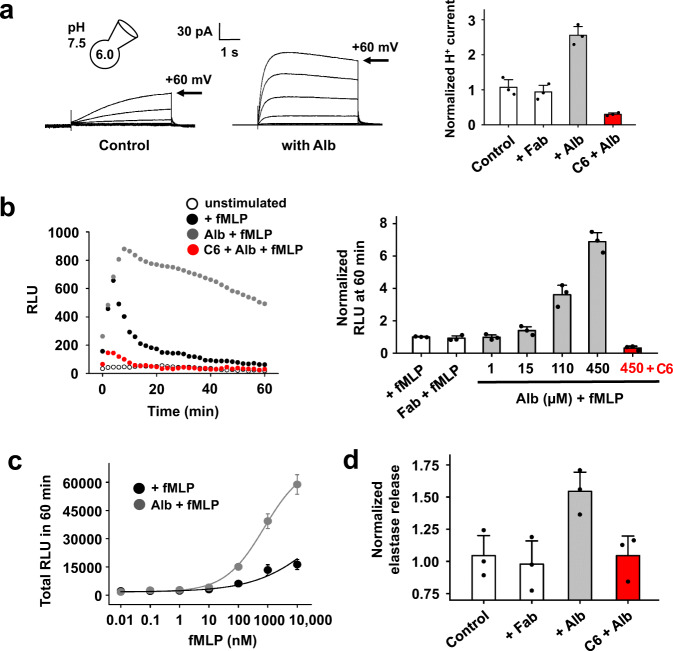
Alb activates hHv1 in human neutrophils to increase ROS production and elastase release. **a** Proton currents passed by hHv1 in human neutrophils studied in the absence (left) and presence (middle) of 450 μM Alb (steps from −60 and +60 mV in 20 mV increments). Fitting activation and deactivation of currents at +60 mV offered time constants (Supplementary Table 1). Right, hHv1 currents measured at the peak of a test pulse to +60 mV after exposing neutrophils to 450 μM Fab (white bar), 450 μM Alb (gray bar), or 450 μM Alb + 20 μM C6 (red bar). Values are normalized to mean current amplitude in the absence of Alb. C6 peptide blocked 87 ± 2% of of the current activated by Alb, *n* = 3 cells. **b** Left, effect of fMLF alone or in combination with Alb on ROS production by human neutrophils. Human neutrophils (2 × 10^5^ cells) were incubated without or with 450 μM Alb, then stimulated with 1 μM fMLF. ROS was measured using luminol (relative light units, RLU). ROS production from unstimulated neutrophils is negligible (white circles). Alb (gray circles) enhanced and sustained the fMLF-stimulated ROS production compared to neutrophils stimulated with fMLF alone (black circles). Incubation with 20 μM C6 (red circles) inhibited ROS production potentiated by 450 μM Alb. Right, ROS produced 60 min after fMLF stimulation. Incubation with Alb increased ROS production by human neutrophils (gray bars); addition of C6 (20 μM, red bar) inhibited the ROS production potentiated by 450 μM Alb. 450 μM Fab had no effect on fMLF-stimulated ROS production (white bar). Values are normalized to mean ROS production by neutrophils stimulated with fMLF alone, *n* = 3 independent experiments. **c** Human neutrophils (2 × 10^5^ cells) were incubated without (black circles) or with 450 μM Alb (gray circles) and then stimulated with increasing concentrations of fMLF. ROS were measured as described in (**b**). Some error bars are smaller than symbols, *n* = 3 independent experiments. **d** Human neutrophils (6 × 10^5^ cells) were incubated with Alb and then stimulated with 1 μM fMLF. Total elastase release at each test condition was measured spectrophotometrically. The values are normalized to elastase release stimulated with fMLF alone (Control, white bar). A 450 μM Alb increased the elastase release by neutrophils (gray), and the increase was inhibited by 20 μM C6 (red) whereas 450 μM Fab had no effect on fMLF-stimulated elastase release (white bar), *n* = 3 independent experiments. Values are mean ± SEM. Source data are provided in the Source Data file.

Here, activation of hHv1 in neutrophils by Alb was demonstrated first by recording native proton currents in freshly-isolated cells from human peripheral blood by whole-cell patch clamp. Application of 450 μM Alb led to a ~2.5-fold increase in native proton currents at +60 mV, with a ~6-fold acceleration of activation and a ~1.5-fold slowing of deactivation, offering an estimated equilibrium affinity (*K*_*d*_) of 112 ± 9 µM (Fig. 2a, left) and a shift of −35 mV in the G-V relationship (Supplementary Fig. 3a and Supplementary Table 1). As observed with sperm, Fab application had no effect on the current and Alb-activated proton currents were suppressed by 20 µM C6, demonstrating their passage by hHv1 (Fig. 2a, right).

To assess ROS release from the neutrophils, we used a luminol-amplified chemiluminescence assay for superoxide anion (O_2_^**·**-^) measurement^35^. Formylated bacterial peptides like fMLF stimulate the production of ROS by neutrophils by binding to G-protein coupled receptors^36^ and triggering intracellular pathways that induce NOX2 to produce O_2_^**·**−4^, ^37^. Here, we observed that 1 µM fMLF stimulated a transient rise and decay in ROS release from neutrophils as expected, whereas the additional presence of 450 µM Alb led to an increase in the peak magnitude of ROS release. More significantly, Alb allowed for sustained ROS production that was still elevated at 60 min, long after release had returned to baseline in the absence of Alb (Fig. 2b, left).

ROS release potentiated by Alb was concentration-dependent across the physiological range, becoming apparent at 15 µM and maintained at a ~7-fold increased rate of release after 60 min with 450 µM (Fig. 2b, right). As expected, neither augmentation of peak ROS release, nor sustained ROS production, was observed with 450 µM Fab, and potentiation was fully inhibited by 20 µM C6 (Fig. 2b, right). In the absence of immune stimulation by fMLF, Alb induced no increase in ROS release above low basal levels, consistent with a role as a potentiator and not the primary stimulus (Supplementary Fig. 3b). Furthermore, Alb potentiation of total ROS production was stimulated with as little as 10 nM fMLF (~1.8-fold) and rose to ~5-fold with 10 µM fMLF without a shift in the fMLF dose-response curve (Fig. 2c, Supplementary Fig. 3c, d).

**Fig. 3:**
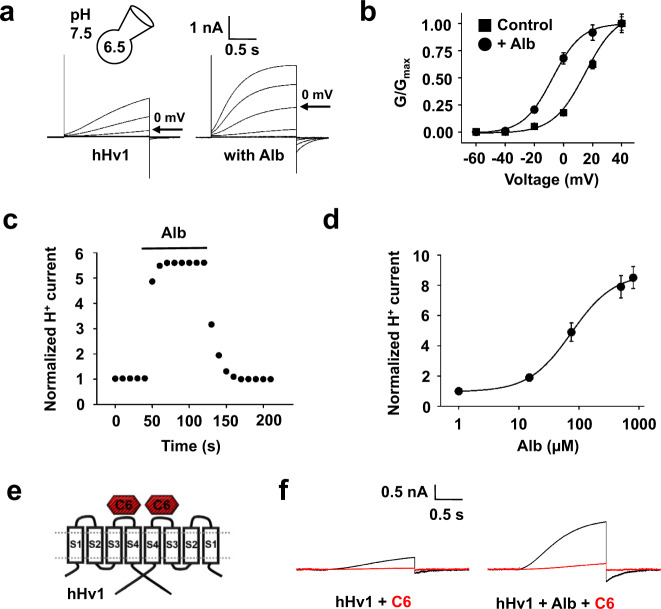
Alb reversibly activates hHv1, shifting activation to more negative voltages. **a** Proton current for hHv1 channels expressed in HEK293T cells before (left), and in the presence of 75 µM Alb (right), with steps of 20 mV from −60 mV to +40 mV. The current measured at the end of depolarization was used to determine the extent of activation. Fitting the activation and deactivation of proton currents at 0 mV to a single exponential function gave time constants τ_act_ of 2619.8 ± 222.3 ms and 332.3 ± 30.6 ms, τ_tail_ of 86.9 ± 8.4 ms and 235.0 ± 29.7 ms without and with Alb, respectively. **b** G-V for hHv1 in the absence (black squares) or presence of 75 µM Alb (black circles). hHv1 channels showed a −23 ± 3 mV shift after exposure to 75 µM Alb from 15 ± 2 mV to −8 ± 1 mV. Curves fit to a Boltzmann equation, *n* = 6 cells. **c** Time course for activation and deactivation of hHv1 currents on acute application (bar) and washout of 75 µM Alb. Currents were recorded at 0 mV. Values are normalized to the control current before the application of Alb. **d** Dose-response relationships for Alb activation of hHv1 at 0 mV. The EC_50_ was estimated from a fit to the Hill relationship to be 74.8 ± 8.7 µM with a coefficient of 1.16 ± 0.11. Values are normalized to mean proton currents amplitude in the absence of Alb, *n* = 6–8 cells. **e** Cartoon showing two C6 peptides binding to an intact dimeric hHv1 channel. **f** C6 peptide (20 μM, red trace) was applied to cells after control pulses (black trace) without Alb (left) or after pre-activation with 75 μM Alb (right). C6 inhibited ~92% of hHv1 proton current (left), which is similar to the extent of inhibition (~89%) with Alb pre-activation (right). Values are mean ± SEM. Some error bars are smaller than symbols. Source data are provided in the Source Data file.

During the inflammatory response, neutrophils also release antimicrobial proteases that cleave bacterial virulence factors, such as elastase^38^. Thus, fMLF-induced neutrophil degranulation can be quantified by measurement of elastase release^21^. Fig. 3d shows that Alb augmented fMLF-stimulated elastase release ~1.5-fold whereas the same amount of Fab had no effect and, demonstrating dependence on H^+^ efflux via hHv1, augmentation was suppressed by 20 µM C6 (Fig. 2d).

hHv1 shows an “enhanced gating mode” during the neutrophil respiratory burst increasing the likelihood of channel opening that is reported to reflect protein kinase C (PKC) phosphorylation of Thr29 at N-terminus of hHv1^39^. Indicating that Alb-activation was not mediated by this mechanism, we observed that hHv1-T29A channels, which are not subject to PKC phosphorylation, were activated by Alb like WT channels (Supplementary Fig. 4).

### Alb also activates cloned hHv1 by changing the voltage-dependence of gating

To explore the mechanism of Alb activation, hHv1 channels were expressed in HEK293T cells and studied by whole-cell patch clamp with a 10-fold proton gradient (pH_i_ = 6.5 and pH_o_ = 7.5), as previously described^5^. In the absence of Alb, hHv1 currents activated slowly in response to depolarization and showed fast deactivation (Fig. 3a, left). When 75 µM Alb was added to the bath solution, hHv1 currents increased ~5-fold due to a ~8-fold acceleration of activation, a ~3-fold slowing of deactivation at 0 mV, and a shift in the V_1/2_ of −23 mV (Fig. 3b and Supplementary Table 1).

Alb stimulation of hHv1 currents was reversible and concentration-dependent with association and dissociation constants of *k*_on_ = 1.3 × 10^3^ ± 0.1 × 10^3^ M^−1^s^−1^ and *k*_off_ = 0.084 ± 0.009 s^−1^ determined by single-exponential fits to the time courses for activation and deactivation, respectively (Fig. 3c). Applying different concentrations of Alb yielded an EC_50_ = 74.8 ± 8.7 µM with a Hill coefficient of 1.16 ± 0.11, consistent with the binding of one Alb per hHv1 dimer (Fig. 3d). Moving sperm from the concentration of Alb in human semen (15 µM) to that in the uterus (500 µM), increased hHv1 currents ~8-fold at 0 mV, shifting the V_1/2_ by −45 mV (Fig. 3d and Supplementary Fig. 5a, b).

The designed peptide C6 inhibits hHv1 by binding to the S3-S4 loops with positive cooperativity, one peptide on each loop (Fig. 3e), thereby biasing the voltage sensors to the down conformation that favors channel closure^5^. As a result, more positive voltages are required to open the channel pores, opening is slowed, and closing is faster; the reverse of the effects of Alb on the channel. Confirming that Alb-induced currents were due to H^+^ permeation through hHv1, 20 µM C6 suppressed nearly all the proton current (Fig. 3f).

This effect of Alb showed specificity and required that the protein was intact, as neither Fab nor Alb subjected to Proteinase K digestion activated hHv1 at concentrations up to 800 µM (Supplementary Fig. 6). Specificity of Alb for hHv1 was further demonstrated by failure of Alb to activate hKv1.3, hKv2.1, hKv1.5, hI_Ks_ (hKv7.1 + hKCNE1), and hNav1.5 (Supplementary Fig. 7) although this represents only a sampling of the many VGICs. Alb activated hHv1 only from the extracellular solution, showing no effect from the cytosol when introduced via the pipette solution (75 µM, *n* = 3 cells), and was observed also to activate hHv1 expressed in *Xenopus* oocytes (Supplementary Fig. 8).

A recent report indicates that a protease-digested form of hHv1 (Hv1Sper) with 68 residues removed from the N-terminus represents as much as half the channel protein isolated from human sperm when visualized by western blotting^40^. We constructed the foreshortened channel by genetic deletion of the residues, expressed it in HEK293T cells and observed the approximately −30 mV shift in the G-V relationship compared to WT hHv1 reported by Berger and colleagues^40^. Indicating that the truncated channels are also sensitive to Alb regulation, we observed application of 500 µM Alb to accelerate activation, slow deactivation, increase current magnitude, and to shift the G-V curve by −15 mV (Supplementary Table 1 and Supplementary Fig. 9).

Because Human Tubal Fluid Medium (HTF, pH 7.2) is routinely used for human IVF^41^ to mimic the native environment, we sought to confirm that the effect of Alb on hHv1 channels was apparent in this solution using a pipette solution at pH 6.7 as estimated for sperm in the human uterus^42^. Indeed, under these conditions, 500 µM Alb increased hHv1 current ~10-fold at 0 mV, shifting the V_1/2_ for conduction by −40 mV (Supplementary table 1 and Supplementary Fig. 10). As a result, the voltage at which hHv1 channels start to open (V_threshold_) shifted from ~0 mV to −30 mV with Alb (Supplementary Fig. 10). These values are comparable to those observed for native proton currents in sperm where Alb produced an approximately −32 mV hyperpolarization in the V_1/2_ and shifted the V_threshold_ ~−30 mV (Fig. 1c).

### Alb binds to the external S3–S4 loop of hHv1

**Fig. 4:**
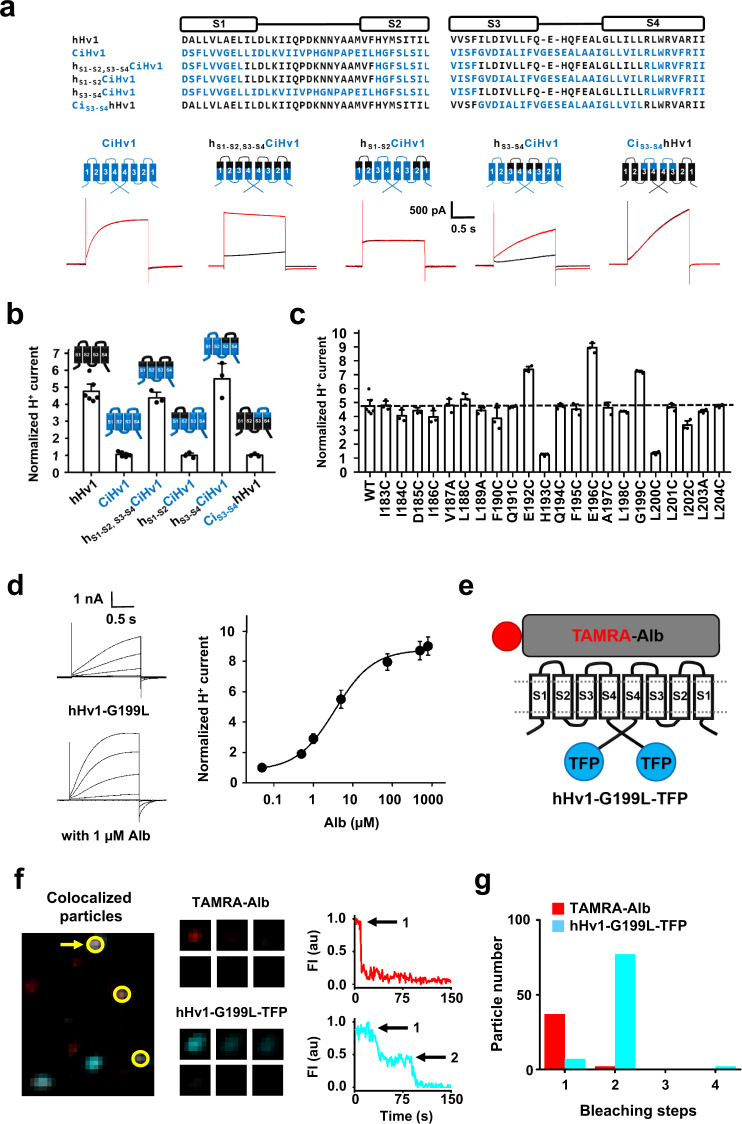
Alb binds to S3-S4 loop of hHv1 channels. hHv1, CiHv1, hHv1-G199L and chimeric channels were expressed in HEK293T cells and studied by whole-cell patch clamp. **a** Top, sequence alignments of the S1-S2 and S3-S4 motifs of hHv1 (black), CiHv1 (blue) and loop-transplant chimeras. Bottom, proton current traces at 0 mV for channels in the absence (black trace) or presence of 75 µM Alb (red trace). **b** Activation by Alb (75 μM) of WT and chimeric channels, current at the end of a test pulse to 0 mV is plotted. Values are normalized to current amplitude before Alb application. The cartoons represent the topology of a single subunit with hHv1 segments in black and CiHv1 segments in blue, *n* = 3–6 cells. **c** Mutagenesis scan of the S3-S4 loop of hHv1 (Cys substitution except for V187A, L189A, and L203A) showing changes with 75 µM Alb at 0 mV; numbering corresponds to the amino acid sequence of hHv1. Values normalized to the current amplitude before Alb application, *n* = 3–6 cells. **d** Left, proton current traces for hHv1-G199L channels in the absence (up) and presence of 1 µM Alb (bottom) with steps of 20 mV from −60 mV to +40 mV. Right, Alb dose-response relationship for hHv1-G199L provides an EC_50_ for Alb activation of 3.4 ± 0.5 µM estimated by a fit to the Hill relationship with a coefficient of 0.93 ± 0.10. Values are normalized to mean proton current amplitude in the absence of Alb. Some error bars are smaller than symbols, *n* = 3–8 cells. **e** Cartoon of one TAMRA-Alb molecule binding to the two subunits in a hHv1-G199L-TFP channel. **f** Left, incubation of TAMRA-Alb (red) with hHv1-G199L-TFP channels (teal) produces single colocalized particles (white) with both TAMRA and TFP fluorescence at the surface of cells (yellow circles). Middle, montage of photobleaching time course of a single fluorescent particle (arrow in left panel) during continuous excitation to bleach the fluorophores (every fifth frame is shown). Right, time courses for simultaneous photobleaching of both fluorophores in the particle, showing one stepwise change in fluorescence intensity for TAMRA-Alb and two for hHv1-G199L-TFP (arrows). **g** Histogram of photobleaching steps for hHv1-G199L-TFP (teal bars) and TAMRA-Alb (red bars) on simultaneous photobleaching. 89% of studied particles with hHv1-TFP were bleached in 2 steps. Data analyzed by the approach of Hines^67^ estimates two hHv1-G199L subunits in surface particles with a confidence of >0.999 (Supplementary Table 2). Among all particles containing both fluorescent colors 95% had one TAMRA-Alb bleaching step at 3 µM TAMRA-Alb (Supplementary Table 3). Values are mean ± SEM. Source data are provided in the Source Data file.

We looked for Alb binding sites on the extracellular portions of hHv1, that is, the residues linking the four membrane-spanning segments, the S1-S2 loop and the S3-S4 loop (Fig. 3e). First, we exchanged the loops in hHv1 with those in CiHv1, a homologous proton channel insensitive to 75 µM Alb, observing when the S1-S2 loop and/or the S3-S4 loops were switched between CiHv1 and hHv1, the channels were functional (Fig. 4a, b).

CiHv1 channels carrying both the S1-S2 and S3-S4 loops of hHv1 (h_S1-S2, S3-S4_CiHv1) were endowed with sensitivity to Alb, whereas transplanting only the S1-S2 loop from hHv1 into CiHv1 (h_S1-S2_CiHv1) was insufficient to confer Alb-activation (Fig. 4a, b). In contrast, transferring only the S3-S4 loop from hHv1 into CiHv1 to create h_S3-S4_CiHv1 conferred Alb sensitivity (*K*_*d*_ ~63 µM) at levels similar to that observed with wild type (WT) hHv1 channels (Supplementary Table 1). hHv1 carrying the S3-S4 loop of CiHv1 (Ci_S3-S4_hHv1) became insensitive to Alb (Fig. 4a, b), supporting the conclusion that the hHv1 S3-S4 loop was the major binding epitope for Alb. Moreover, transplanting the S3-S4 loop of hHv1 into the hKv2.1 potassium channel conferred weak potentiation by Alb (Supplementary Fig. 11), supporting the notion that the mechanism of Alb action was via the voltage sensor loop.

To explore the role of the hHv1 S3-S4 loop in Alb binding, we carried out a mutational scan where loop residues I183 to L204 were changed individually to Cys and the response of the mutant channels to Alb was studied (Fig. 4c). Two mutations, hHv1-H193C and hHv1-L200C, fully eliminated Alb activation, suggesting that these two residues may mediate direct interaction with Alb. Three other changes (hHv1-E192C, hHv1-E196C, and hHv1-G199C) enhanced Alb activation. Studying these sites with further mutations yielded hHv1-G199L, a proton channel with a ~22-fold improvement in the Alb EC_50_ (3.4 ± 0.5 µM) compared to WT hHv1 and no change in the G-V relationship (Fig. 4d and Supplementary Table 1). Furthermore, fitting the dose-response curve of Alb on the channel yielded a Hill coefficient of 0.93 ± 0.10 like WT channels, consistent with binding of single Alb. The G199L mutant accelerated Alb association ~5-fold (*k*_on_ = 6.9 × 10^3^ ± 0.7 × 10^3^ M^−1^s^−1^) and slowed dissociation ~4-fold (*k*_off_ = 0.022 ± 0.003 s^−1^).

### One Alb binds to one dimeric hHv1 channel

The increased affinity of Alb for hHv1-G199L channels allowed a direct study of binding using smTIRF and live cells (Methods). Alb was labeled with carboxytetramethylrhodamine (TAMRA) on its N-terminus to yield TAMRA-Alb and the channel was tagged on its C-terminus with a teal fluorescent protein to produce hHv1-G199L-TFP (Fig. 4e). Incubation of TAMRA-Alb with HEK293T cells expressing hHv1-G199L-TFP produced doubly-labeled particles containing both fluorescent tags at the cell surface (Fig. 4f, left). Cells subjected to intense illumination were used to count the number of Hv1-G199L-TFP subunits and TAMRA-Alb molecules by single molecule fluorophore bleaching, as previously described^5^.

Control experiments with hHv1-G199L-TFP subunits showed two stepwise decreases in fluorescence in 89% of particles (Fig. 4f, g and Supplementary Table 2), as expected from hHv1 dimeric assembly. We report few, if any, monomer channels on the cell surface (Supplementary Table 2)^43^, as the observed singleton events were consistent with prebleaching and missed events based on the bandwidth of the smTIRF system^5^.

When 3 µM TAMRA-Alb was applied to cells expressing hHv1-G199L-TFP, smTIRF microscopy yielded a mean Manders’ coefficient for colocalization (MCC) of 0.50 ± 0.06, suggesting that roughly half the channels were associated with TAMRA-Alb (Supplementary Table 3). Of the hHv1-G199L-TFP particles directly confirmed to be intact dimeric channels (that is, showing two TFP bleaching steps) and to be colocalized with TAMRA-Alb, 95% were observed to be associated with one Alb, and 5% were visualized with two (Fig. 4f, g and Supplementary Table 3). Binding of one Alb per dimeric hHv1 channel is reasonable given its dimensions estimated from 3D structures (30 × 80 × 80 Å^3^, PDB 1BM0)^16^, far exceeding the exposed surface of the dimeric hHv1 channel (~ 40 × 40 Å^2^)^43^.

As an independent test of the 1 Alb to 1 dimeric hHv1 channel stoichiometry, we studied monomeric hHv1 (ΔhHv1), engineered by shortening the N-terminus and removing the C-terminus coiled-coil domain, as described^5^. Supplementary Figure 12 shows that while ΔhHv1 channels were operational, they did not respond to 75 µM Alb. We suspect our observation that high Alb concentrations were able to activate ΔhHv1 with an EC_50_ = 1,904 ± 155 µM and a Hill coefficient of 1.05 ± 0.06 (Supplementary Fig. 12b), reflects the response of dimeric channels that form as a minor population with these truncated subunits^43^.

### Alb DII is the principal domain for binding to hHv1

**Fig. 5:**
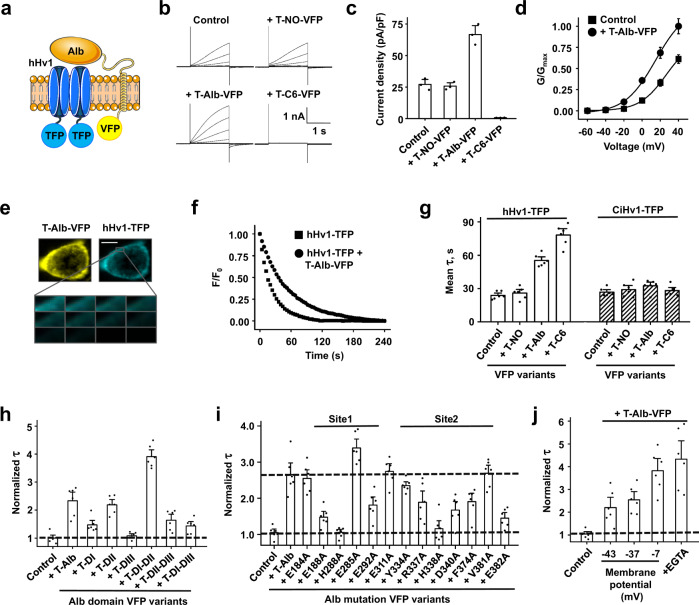
DII of Alb is required for binding to hHv1 channels. Tethered constructs carrying VFP were expressed in HEK293T cells with channels tagged with TFP and studied by FRET microscopy. **a** Schematic representation of T-Alb-VFP showing binding of Alb to the hHv1-TFP channel. Extracellular interaction of Alb and hHv1 brought intracellular TFP and VFP into close proximity (<10 nm), resulting in FRET that was quantified by measuring the mean time constant (τ) for TFP (donor) photobleaching. **b** Representative current traces (20 mV steps from −60 to +40) for hHv1-TFP channels expressed alone (Control) or with T-Alb-VFP, T-NO-VFP (no Alb inserted), or T-C6-VFP. **c** Whole-cell peak current densities of hHv1-TFP channels at 0 mV expressed alone (Control) or with T-NO-VFP, T-Alb-VFP, or T-C6-VFP studied as in (**b**), *n* = 3 cells. **d** G-V relationships for hHv1-TFP expressed alone (black squares) or with T-Alb-VFP (black circles). Curves are fit to a Boltzmann equation, *n* = 3 cells. **e** hHv1-TFP (cyan) and T-Alb-VFP (yellow) are shown to reach the cell surface. Scale bar is 10 μm. The boxed area of membrane shows donor photobleaching with continuous illumination. **f** Exemplar photobleaching showing the decay of fluorescence for regions of single cells expressing hHv1-TFP (black squares) or hHv1-TFP with T-Alb-VFP (black circles). τ was determined by single-exponential fits to the time course of photobleaching. **g** FRET showing hHv1-TFP (white bars) assembly with T-Alb-VFP or T-C6-VFP but not T-NO-VFP. CiHv1-TFP (white bars with black slash)^5^, did not show FRET with T-Alb-VFP or T-C6-VFP, *n* = 6 cells. **h**, Tethered Alb domain variants expressed with hHv1-TFP for FRET studies. τ of photobleaching for each pair was normalized to the τ of photobleaching of hHv1-TFP alone (Control). *n* = 6 cells. **i** T-Alb-VFP point mutations designed based on the two predicted interaction sites (Supplementary Fig. 13), expressed with hHv1-TFP for FRET studies. Control is hHv1-TFP expressed alone, *n* = 6 cells. **j** Adjusting KCl in the bath by isotonic substitution for NaCl from 2 mM to 10 mM and 100 mM changed the HEK293T cell RMP from −43 ± 6 mV, to −37 ± 4 mV and −7 ± 2 mV, respectively. Increased binding of T-Alb-VFP and hHv1-TFP with membrane depolarization as demonstrated by the increasing τ, from 52 ± 7 s to 60 ± 7 s and 90 ± 11 s. When 1 mM EGTA was added to the solution with 10 mM KCl τ was further increased to 101 ± 15 s. Control is hHv1-TFP expressed alone with 10 mM KCl, *n* = 6 cells. Values are mean ± SEM. Source data are provided in the Source Data file.

To facilitate characterization of the Alb-hHv1 interaction by mutational screening, we constructed a gene allowing expression of Alb variants on the extracellular surface of cells via a membrane tether. The construct was comprised by the nucleotides encoding Alb (or Alb variants) in-frame with the code for the transmembrane helix of the platelet-derived growth factor receptor carrying the fluorescent protein mVenus (VFP) on its intracellular C-terminus to produce T-Alb-VFP (“Methods”)^44^. This permitted study of the interaction of T-Alb-VFP constructs and hHv1 tagged with teal fluorescent protein on its C-terminus (hHv1-TFP) in live cells (Fig. 5a) using FRET microscopy^45^.

When T-Alb-VFP was expressed with hHv1-TFP, it potentiated the channel to a similar extent as soluble Alb. T-Alb-VFP increased current density at 0 mV ~3-fold, speeding the kinetics of activation ~4-fold, slowing deactivation ~2-fold, and shifting V_1/2_ by −12 mV, values consistent with an effective concentration of free Alb in solution of ~35 µM (Fig. 5b–d). Two control tethered constructs, one with C6 peptide (T-C6-VFP) and another with no Alb insert (T-NO-VFP), further validated the approach, producing full inhibition and no potentiation, respectively (Fig. 5b, c). This data is consistent with our prior use of other tethered toxins and channels^46^.

The physical interaction of T-Alb-VFP with hHv1-TFP at the membrane surface of HEK293T cells was measured using the photobleaching rate of the TFP donor (Fig. 5e). hHv1-TFP alone, and hHv1-TFP expressed with T-NO-VFP, showed a mean time constant (τ) for photobleaching of 24 ± 4 s and 27 ± 5 s, respectively (Fig. 5f, g). Demonstrating direct interaction of hHv1-TFP and T-Alb-VFP, the τ for hHv1-TFP increased to 54 ± 6 s, on expression with T-Alb-VFP, and to 78 ± 8 s on expression with T-C6-VFP (Fig. 5f, g). Consistent with the failure of Alb to activate CiHv1, no FRET was observed between CiHv1-TFP and T-Alb-VFP (Fig. 5g).

Alb consists of three homologous domains: DI (residues D1 to R197); DII (residues L198 to Q385), and DIII (residues N386 to L585)^15,47^. We investigated how each domain contributed to hHv1 binding by developing six tethered Alb domain variants, T-DI-VFP; T-DII-VFP; T-DIII-VFP; T-DI-DII-VFP; T-DII-DIII-VFP; and T-DI-DIII-VFP (Methods). When the interaction between tethered Alb domains and hHv1 channels on the cell surface were measured by FRET, the variant consisting Alb DII alone (T-DII-VFP) interacted with hHv1-TFP like WT Alb (T-Alb-VFP) (Fig. 5h). In contrast, DI alone (T-DI-VFP) appeared to interact weakly with hHv1-TFP, and T-DIII-VFP showed no evidence for a role in binding to the channel (Fig. 5h). Supporting the notion that DIII was redundant, the DI-DII variant (T-DI-DII-VFP) showed an even stronger interaction than WT Alb, while T-DII-DIII-VFP showed less FRET with hHv1-TFP than DII alone (Fig. 5h). Corroborating an essential role for DII, the variant lacking that domain (T-DI-DIII-VFP) displayed greatly reduced FRET with hHv1-TFP (Fig. 5h).

In the absence of high resolution information for the Alb-hHv1 complex, we performed an in silico docking analysis (HPEPDOCK)^48^ using the crystal structure of Alb (PDB 1BM0)^16^ and the 11-residue binding epitope in S3-S4 loop of hHv1 (F190 to L200). Initial docking predicted two sites in Alb, separated by ~27 Å, as putative hot spots for interaction (Supplementary Fig. 13a, c). This distance is comparable to that between hHv1-H193 in the two hHv1 channel subunits (~26 Å) in the structural model of hHv1 based on electron paramagnetic resonance (EPR) spectroscopy^43^. Consistent with the loss-of-function mutation that fully-eliminated Alb activation (Fig. 4c), hHv1-H193 appears to be a key component of the Alb “receptor” in the S3-S4 loop. Two low energy poses from in silico docking exhibited interaction between hHv1-H193 and Alb-E188, Alb-H288, Alb-E292 in Site 1 (Supplementary Fig. 13b). Three low energy poses exhibited interaction between hHv1-H193 and Alb-Y334, Alb-R337, Alb-H338, Alb-D340, Alb-F374, Alb-V381 in Site 2 (Supplementary Fig. 13d). These predicted interacting residues are in Alb DII, with the exception of E188 in DI, an observation supported by our FRET data (Fig. 5h).

We tested the docking predictions by generating eleven T-Alb-VFP variants, three in Site 1 (E188A, H288A, E292A) with nearby E184A as a control, and six in Site 2 (Y334A, R337A, H338A, D340A, F374A, V381A) with nearby E311A as a control. Each T-Alb-VFP mutant was evaluated for its impact on the interaction with hHv1-TFP in live cells using FRET (Fig. 5i). In Site 1, the mutation H288A showed the largest effect on Alb binding; mutants E188A, E292A had moderate effects, and nearby E184A did not alter Alb binding. In Site 2, H338A had the largest effect; Y334A, R337A, D340A, and F374A had moderate effects, and both V381A and nearby E311A did not alter the binding (Fig. 5i). Thus, Alb residues H288 in Site 1 and H338 in Site 2, both in Alb DII, appeared to be fundamental to binding to hHv1.

### A proposed model of Alb binding to hHv1

**Fig. 6:**
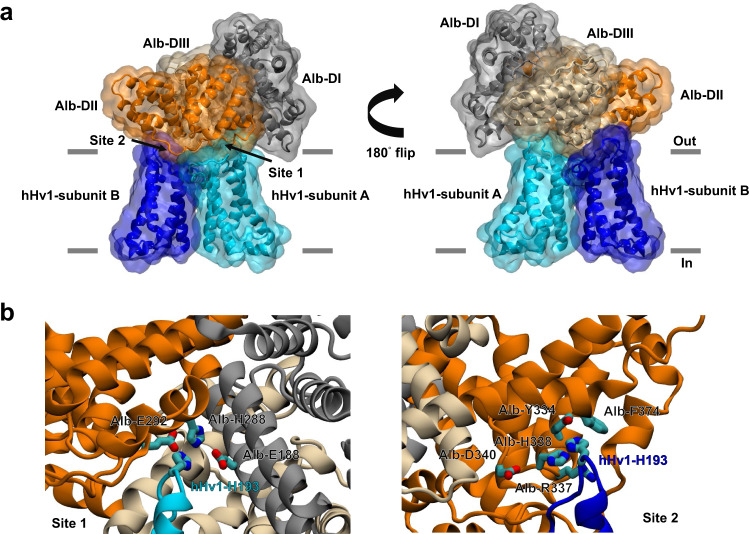
A proposed Alb-hHv1 structural model from molecular dynamics simulations. **a** The Alb-hHv1 complex in two orientations showing the three Alb domains DI (gray), DII (orange) and DIII (wheat) and the two subunits (cyan and blue) of a dimeric hHv1 channel. The horizontal gray lines indicate the position of the membrane boundary. **b** Close-up view of the interaction interface between Alb and two individual hHv1 subunits in two binding sites in the structural model. Left, interaction Site 1, H193 of hHv1 subunit A (cyan) may form direct interaction with Alb residue H288 and the adjacent E188 and E192. Right, interaction Site 2, H193 of hHv1 subunit B (blue) may form direct interaction with Alb residue H338 and the adjacent Y334, R337, and F374.

A structural model of the Alb-hHv1 complex was generated from MD simulations using the NAMD program^49^, based on our knowledge of the interfacial residues identified as critical for binding by patch-clamp (Fig. 4c) and FRET (Fig. 5i) experiments, the crystal structure of Alb (PDB 1BM0)^16^, and the EPR-derived structural model of hHv1^43^ (Fig. 6a and Supplementary Fig. 14). An all-atom model of the transmembrane region of the dimeric hHv1 channel (residues G90 to I218) was embedded in a lipid bilayer with excess hydration. An Alb molecule was placed in the extracellular solution with the Alb-DII domain facing the extracellular surface of the channel (Supplementary Fig. 11). At the beginning of the simulation, the Alb molecule was pulled slowly toward hHv1 with distance restraints between centers of mass of side-chain heavy atoms of the highest-impact residues, that is, hHv1-H193 in each of channel subunit and Alb-H288 and Alb-H338, in Sites 1 and 2, respectively. These restraints with a target distance of 5 Å were gradually applied over 10 ns using the Colvars module in NAMD. The local residue-residue interactions in Site 1 (containing hHv1-H193 of subunit A and Alb-E188, Alb-H288, and Alb-E292), Site 2 (involving hHv1-H193 of subunit B and Alb-Y334, Alb-R337, Alb-H388, and Alb-F374), and other contact regions of the two proteins were refined in subsequent 150 ns simulations (Supplementary Table 4). Finally, 1.5 µs long timescale ANTON2 simulation^50^ was performed to further relax the Alb-hHv1 complex (Supplementary Fig. 15, 16 and 17).

The final Alb-hHv1 model reveals specific interactions (Fig. 6a) consistent with the experimental observations from scanning mutagenesis of the channel (Fig. 4c) and Alb (Fig. 5i). Thus, the model exhibits electrostatic interactions in Site 1 between hHv1-H193 (subunit A) and Alb-E188 and Alb-E292; and π-π stacking interaction between hHv1-H193 and Alb-H288 (Fig. 6b, left). In Site 2, hHv1-H193 (subunit B) interacts with Alb-Y334, Alb-R337, Alb-H338 via electrostatic and hydrogen bonding interactions and with Alb-F374 via the CH-π or π-π stacking interaction (Fig. 6b, right). Here, Alb-D340 is ~10 Å away from hHv1-H193 (subunit B), while the mechanism underlying the disruptive effect of its mutation (Fig. 5i) requires more investigation, one explanation could be that its electrostatic interaction with Alb-H338 is important to the interaction of Alb-H338 and hHv1-H193.

hHv1-L200 was found to influence Alb activation (Fig. 4c), but is predicted to be buried in the membrane at resting state^4,43^ and did not appear to be directly involved at the interaction interface in our resting state model; we suspect that mutation might change the structure of Alb-hHv1 complex in a disruptive manner as a consequence of its location in the activated channel state.

Two experiments inspired by the MD simulation results were performed and further supported the model of the Alb-hHv1 complex. First, an unanticipated hydrogen bond between Alb-E382 near Site 2 and hHv1-Y134 (subunit B) was observed in the model. When the Glu residue was neutralized by mutation to alanine (producing T-Alb-E382A-VFP), the binding affinity of Alb to hHv1-TFP assessed by FRET was reduced (Fig. 5i). Second, the model identified hHv1-E192 in subunit A to be within 4 Å of negatively-charged Alb-E285. Neutralizing this acidic residue in Alb (Alb-E285A-VFP) improved binding with WT hHv1 (Fig. 6i). The model further rationalized mutagenesis data that had shown neutralizing hHv1-E192 increased activation by WT Alb (Fig. 4c) by removing the destabilizing electrostatic repulsion with Alb-E285.

Consistent with the notion that Alb binds to the hHv1 VSDs to activate the channels, the binding affinity of Alb was observed to increase with membrane depolarization as judged by FRET. Thus, the proton channel VSDs move “outward” to the active conformation in response to increased membrane potential^4,5^ and we observe that the affinity of the interaction of T-Alb-VFP and hHv1-TFP increased as the resting membrane potential (RMP) was increasingly depolarized from −43 ± 6 mV to −7 ± 2 mV by isotonic replacement of NaCl with KCl in the bath solution (Fig. 5j).

## Discussion

### Essential role of Alb in human sperm capacitation

Two outstanding questions in sperm physiology are answered by our identification of Alb activation hHv1 channels as central to initiation of capacitation. First, the RMP of non-capacitated sperm is estimated to be −20 mV to −40 mV^51^, whereas the threshold for opening of hHv1 channels was reported to be ~0 mV in the ionic environment present in the uterus^4,52,53^. This presented an enigma, how did hHv1 open under natural conditions? Here, we answer to the conundrum; because the uterus contains ~500 µM Alb, the V_threshold_ for hHv1 shifts to ~ −30 mV (Supplementary Fig. 10). Whereas Alb concentration is too low in semen to activate hHv1 prematurely, the level is high enough in the female reproductive tract to activate the channel and increase H^+^ efflux, leading to intracellular alkalization and capacitation (Fig. 1, Supplementary Figs. 1 and 10)^5,7^. Second, our findings offer a mechanistic rationale for the increase in pregnancy rates with IVF on supplementation with Alb^19,20^, one that does not comport with two prior hypotheses for the mechanism by which Alb might enhance oocyte fertilization.

Alb has been proposed to extract cholesterol from the sperm plasma membrane thereby allowing HCO_3_^−^ influx to alkalinize the cells^54^, apparently obviating a role for H^+^ flux via hHv1 that we demonstrated using C6 to block the channel^5^. Here, we observe that when Alb is pre-saturated with cholesterol it induces the same increase in pH_i_ in non-capacitated human sperm as Alb alone (Supplementary Fig. 1g). Furthermore, Alb pre-saturated with cholesterol activates hHv1 channels in HEK293T cells like Alb alone (Supplementary Fig. 18a), indicating that even when Alb is unable to sequester additional cholesterol it can activate the channel to achieve sperm alkalization. We support an alternative explanation for the loss of cholesterol from sperm during capacitation. Since the equilibrium level of HCO_3_^-^ in cells is a function of total carbon concentration and pH^55^, alkalization caused by increased H^+^ efflux via hHv1 will increase intracellular HCO_3_^-^ and this has been demonstrated by others to activate adenylyl cyclase and protein kinase A, upregulating phospholipid scramblases that facilitate cholesterol loss from sperm^27,56^.

A second hypothesis for augmented fertilization success with Alb has been that it sequesters Zn^2+^, decreasing the already low concentration of Zn^2+^ in the uterus compared to semen^57^, thereby releasing hHv1 from residual Zn^2+^ inhibition^7^. While such a role for Alb remains possible, we observe that Alb has similar stimulatory effects on hHv1-H140A and WT hHv1 channels (Supplementary Fig. 18b, c), although the mutant has an ~30-fold lower affinity for Zn^2+^ than WT channels^2^. This shows that hHv1 activation by Alb does not require Zn^2+^ chelation.

Furthermore, hHv1 activation by Alb does not appear to result from chelation of trace elements. It has become commonplace to add EDTA or EGTA to bath solutions for biophysical studies of hHv1 because this speeds current activation, slows deactivation, and shifts the G-V relationships to more hyperpolarized potentials, increasing the current magnitude so the channel is easier to study^4^. The effects of EDTA and EGTA have been attributed to chelation of trace heavy metals contaminating salt solutions^4^. We do not support the notion that Alb activates hHv1 by chelation of trace metals, first, because Alb activation is increased (or decreased) by mutating sites on the channel (Fig. 4c) or Alb (Fig. 5i) that mediate Alb-hHv1 binding. Further, we presented two examples of mutations at the Alb-hHv1 binding interface that are suppressed by secondary mutations on the other protein (hHv1-Y134 and Alb-E382; hHv1-E192, and Alb-E285, Fig. 6i) and it seems unlikely that each mutation, and restoring counter mutation, would modify heavy metal binding by Alb especially those on the channel. Finally, the hyperpolarizing shift in the V_1/2_ of hHv1 in sperm induced by EDTA is far less than that produced by Alb. Thus, the V_1/2_ in the absence of both EDTA and Alb is ~15 mV, addition of 500 μM Alb produces a negative shift of 53 mV to −38 mV, whereas 1 mM EDTA alone produces a shift only to 4 mV (Supplementary Table 5, and Supplementary Fig. 19); further suggesting that they act differently, adding both Alb and EDTA produces a smaller shift, to V_1/2_ = −28 mV, than Alb alone.

Similar to what we observed in sperm, 500 μM Alb alone shifts the V_1/2_ of cloned hHv1 channels in HEK293T cells −45 mV (Supplementary Table 5). However, in contrast to sperm, EDTA and EGTA equally effective as Alb on the heterologously-expressed channel, inducing a shift approximately −40 mV. Of note, the interaction of T-Alb-VFP and hHv1-TFP measured by FRET increases on application of 1 mM EGTA, in the same fashion as membrane depolarization (Fig. 5j). This suggests that EGTA, like Alb, favors the outward configuration of the hHv1 VSDs to increase Alb binding, shifting the V_1/2_ so far that Alb has no further effect (Supplementary Fig. 20). The partial impact of EGTA/EDTA on sperm H^+^ current compared to Alb (versus the homologous impact on the cloned channel in HEK293T cells) suggests we do not fully understand how the chemical chelators alter hHv1 function and invites further exploration.

**Fig. 7:**
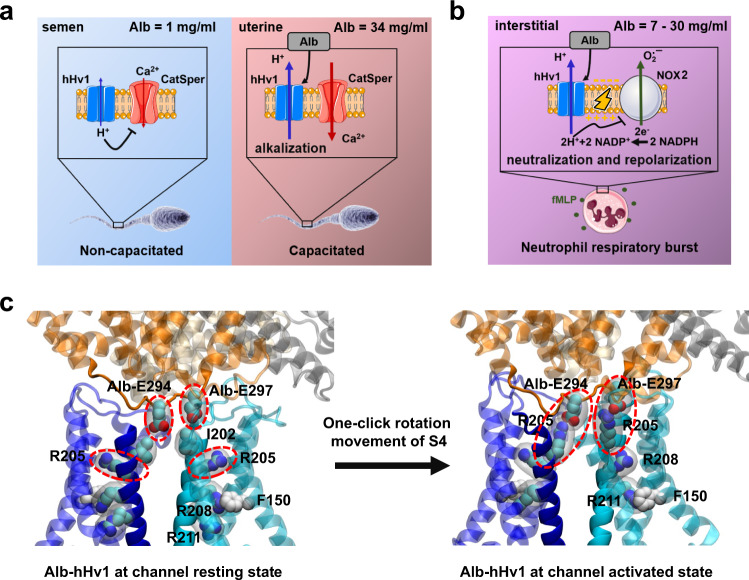
Physiology of Alb activation of hHv1 in human sperm and neutrophils and proposed molecular mechanism. **a** The higher level of Alb in the female reproductive tract activates hHv1 in sperm leading to H^+^ efflux and cytosolic alkalization, initiating capacitation by potentiating CatSper. The synergy of hHv1 and CatSper in human sperm capacitation has previously been described^5–7^. **b** Activation of hHv1 in neutrophils by Alb potentiates H^+^ efflux, maintaining physiological pH_i_ and compensating for the depolarization caused by NOX2, enhancing enzyme activity and allowing sustained ROS production. During the neutrophil respiratory burst (here, stimulated by fMLF), NOX2 translocates electrons from NADPH across the membrane to reduce O_2_ to O_2_·^-^; this efflux of electrons generates H^+^ in the cytoplasm and depolarizes cells, inhibiting the activity of NOX2^5,8–10^. **c** Alb activation of hHv1 is proposed to proceed by an electrostatic gating modification mechanism. Two binding sites in the Alb DII domain interact with the two S3-S4 loops in hHv1 channels. The two Alb sites each have an adjacent negatively-charged residue (Alb-E294 and Alb-E297) that attracts the first channel gating charge (hHv1-R205) to facilitate outward movement of the voltage sensors and stabilizes the sensors in the activated “up” conformation. Side chains of three gating charges (R205, R208, and R211), the “hydrophobic gasket” formed by F150 that separates the internal and external aqueous solutions^73^, and I202, one helical turn above R205, are shown on hHv1 subunit A (cyan).

Alb potentiation of hHv1 is established here as a required and natural mechanism for capacitation initiation by three observations (Fig. 7a). First, our prior demonstration that block of hHv1 by C6 inhibits capacitation (Fig. 1 and Supplementary Fig. 1)^5^. Second, our demonstration here that direct binding of Alb to hHv1 shifts the V_threshold_ so the channel is active at the hyperpolarized membrane potentials measured in human sperm (Figs. 1, 3 and Supplementary Fig. 3, 10). And, third, that Alb concentration in the uterus, but not semen, is sufficient to activate hHv1 (Fig. 1).

### Alb modulates the innate immune system via hHv1

Neutrophils are the most abundant white blood cell type and an essential part of the innate immune system that defends against invading pathogens via ROS release, a process that is also implicated in pathogenesis related to oxidative stress^32^. When stimulated, neutrophil NOX2 produces ROS by reducing O_2_ to O_2_^**·**−^, a reaction that translocates electrons across the plasma membrane, depolarizing cells, and generating protons in the cytoplasm that inhibit further NOX2 activity^23,33^. Proton efflux via Hv1 allows sustained ROS production by NOX2 by maintaining appropriate membrane potential and pH_i_^8,10^, as demonstrated by genetic deletion of Hv1 in mice^9^ and by C6 blockade in human neutrophils^5^.

In this study, we used fMLF to induce the respiratory burst in human neutrophils and show that Alb acts via hHv1 to enhance NOX2 activity. Indeed, Alb was previously observed to increase ROS production by neutrophils although the relevant receptor and mechanism were unknown^22^. The importance of Alb potentiation of hHv1 in neutrophils can be inferred from estimates that during the respiratory burst the cells would depolarize from roughly −60 mV^58^ to +200 mV within 20 ms without charge compensation by hHv1^34^, and that activation of hHv1 is slower in the absence of Alb (Figs. 2a and 3a). Moreover, it is projected that NOX2 activity without offset would decrease pH_i_ by more than five units based on the amount of O_2_^**·**−^ generation^59^. Potentiated by Alb, hHv1 opens at more negative membrane potentials and faster allowing for more effective compensation. Of note, as the concentration of stimulatory fMLF is increased, the potentiating effect of Alb increases to meet the higher demand for compensation (Fig. 2c, Supplementary Fig. 3d).

A number of the attributes of the neutrophil response appear to protect against aberrant inflammatory mediator release. Alb in the absence of a pathogenic stimulus does not induce ROS production (Supplementary Fig. 3b). Furthermore, the level of Alb varies in different tissues^14^ but it averages ~30 mg/mL (450 µM) in many compartments. This Alb concentration will shift the V_threshold_ of hHv1 approximately −30 mV (Supplementary Fig. 3b), therefore is estimated to bring the neutrophil hHv1 V_threshold_ in native environment (pH_i_ = 7.3, pH_o_ = 7.4) from ~20 mV to −10 mV, a level still far above the RMP of approximately −60 mV^58^ until immune stimulation produces a respiratory burst that depolarizes the cells. Indeed, in many tissue compartments Alb is low in health but increases with disease, for example, the hallmark of infectious acute respiratory distress syndrome in the lung is accumulation Alb-rich edema and neutrophilic infiltrates in the air-spaces^60^. The presence of Hv1 in mouse neutrophils has been shown to facilitate Ca^2+^ influx^10^, and this is required for elastase release^61^. This may explain why the action of Alb on hHv1 also increases elastase release from human neutrophils (Fig. 2d). Our findings demonstrate that Alb is an innate immune system regulator and identifies potentiation of hHv1 as a previously unrecognized innate immunity mechanism that enhances the inflammatory activity of neutrophils in health and disease (Fig. 7b).

### A physical model for Alb binding on hHv1

Given the critical physiological roles demonstrated for Alb via hHv1, and in the absence of high resolution atomic structural information, we judged it valuable and feasible to construct a structural model of the Alb-hHv1 macromolecular complex. The Alb-hHv1 model was constructed by MD simulations (Fig. 6a), and evaluated experimentally using Alb and Alb variants tethered to the extracellular surface of the cells expressing hHv1 or hHv1 variants (Fig. 5h, i), a strategy previously applied to peptide neurotoxins to identify residues important in toxin-channel complexes^46^. The model locates hHv1-H193 of subunit A in Site 1 with Alb-H288, and hHv1-H193 of subunit B in Site 2 with Alb-H338 (Fig. 6b). These interactions appear to coordinate Alb binding to the two voltage-sensor loops in hHv1 channels (Fig. 6a, b), while other Alb residues (E188, E292, Y334, R337 and F374) contribute to the two sites through a complex network of residue-residue interactions (Fig. 6b). The model rationalizes why neutralizing hHv1-E192 in the channel improves WT Alb activation (Fig. 4c). We consider limitations of our modeling approach, including the persistence of restraints during long-term simulations and our inability to simulate the complete binding/unbinding process due the large size of the system (~185,000 atoms) in Supplementary Fig. 14.

### A proposed mechanism, Alb as an electrostatic gating modifier

Some neurotoxins target VGICs to activate or inhibit the channels by favoring the “up” or “down” state of the voltage sensors, respectively. By this mechanism CssIV activates Nav1.2, while C6 inhibits hHv1^5,62^. We suggest that the physiological effects of Alb on hHv1 take place by a similar mechanism, whereby Alb favors the activated position of the two hHv1 voltage sensors.

hHv1 responds to membrane potential changes via three arginine residues, R205, R208 and R211 in the S4 helix (Fig. 7c)^4^. The residues move in the electric field facilitated by interactions with acidic residues in the neighboring S1–S3 helices^13^. Recently, a µs timescale MD simulation of hHv1 suggested that depolarization moves the S4 helix upward, placing R205 at the polar-apolar membrane surface near D123 in the S1 helix^63^. In our model of the hHv1 resting state, the S4 helices are in the “down” position, placing hHv1-I202 (one helical turn above the first gating charge hHv1-R205) in channel subunits A and B toward Alb-E294 and Alb-E297 of Alb DII, respectively. We posit that upon depolarization, the electrostatic attraction between the two hHv1-R205 residues and the two negative Alb residues, Alb-E294 and Alb-E297, lowers the energy for the upward movement of the S4 helices, accelerating and sustaining the activation of hHv1 (Fig. 7c).

Supporting the notion that Alb acts as a gating modifier, it shifts the activation of hHv1 to more negative potentials (Fig. 3b and Supplementary Fig. 5b) and speeds channel activation (Fig. 3a and Supplementary Fig. 5a). Also supporting this mechanism, the affinity of Alb for hHv1 was observed to increase with depolarization (Fig. 5j and Supplementary Fig. 5c). Finally, we highlight the fact that neutralizing the channel residue to produce hHv1-R205N yields a channel that is insensitive to Alb (Supplementary Fig. 21a), whereas hHv1-R211S channels were activated like WT (Supplementary Fig. 21b). This is in agreement with the molecular mechanism we propose based on a closed state model where hHv1-H193 in each subunit interacts with Alb-H288 or Alb-H338, and an open state model where the “up” sensor position allows the first gating charge in each channel subunit (hHv1-R205) to interact with Alb-E294 and Alb-E297, favoring the active channel conformation and potentiated proton fluxes.

hHv1 is implicated in a wide range of biological processes in addition to the capacitation of sperm and the innate immune responses we study here^4^. The channels have some notable roles in proliferation of cancer cells^24^, tissue damage during ischemic stroke^64^, and hypertensive injury of the kidney^65^. Because Alb is ubiquitous at levels that vary in different human compartments in health and disease^14^, the potentiation of hHv1 by Alb we describe in this report will be wide-spread, tissue-dependent, and play both salutary and unfavorable roles in human physiology.

## Methods

### Molecular biology

The coding sequences for human Hv1 (NM_001040107) or *Ciona intestinalis* Hv1 (NM_001078469) were tagged with a teal fluorescent protein (TFP) via a 13-residue linker using gBlock gene fragments (Integrated DNA Technologies) and inserted into a laboratory dual-purpose vector pMAX using Gibson Assembly (New England BioLabs). h_S1-S2, S3-S4_CiHv1 was constructed by replacing the nucleotides for the S1-S2 loop (L169-L187) and S3-S4 loop (G231-L254) of CiHv1 with S1-S2 loop (I121-F139) and S3-S4 loop (I183-L204) of hHv1. Similarly, h_S1-S2_CiHv1 was constructed by replacing the S1-S2 loop (L169-L187) of CiHv1 with S1-S2 loop (I121-F139) of hHv1; h_S3-S4_CiHv1 was constructed by replacing the S3-S4 loop (G231-L254) of CiHv1 with S3-S4 loop (I183-L204) of hHv1; and, Ci_S3-S4_Hv1 was constructed by replacing the S3-S4 loop (I183-L204) of hHv1 with S3-S4 loop (G231-L254) of CiHv1. All chimeras were assembled into pMAX using Gibson assembly. Hv1Sper was constructed by truncating 67 residues at the N-terminus of hHv1 (residues from A2 to R68). hKv1.3 (NM_002232), hKv1.5 (NC_000012), hKv2.1 (NP_004966), hKCNQ1 (NM_000218), hKCNE1 (NP_000210.2), hNav1.5 (NC_000003) and the sodium voltage-gated channel β subunit 1 (NC_000019) in pMAX were used as previously described. Tethered Albumin (Alb) constructs were built in pMAX vector. To generate T-Alb-VFP, the PDGF-receptor transmembrane domain was amplified and inserted into pMAX vector. Subsequently, sequences encoding the preprotrypsin secretory pathway signal sequence (secretion signal), Alb sequence and a 16 residues flexible linker region (Gly-Asn) x 8 were inserted at the 5ʹ end of the PDGF transmembrane domain. Fluorescent protein mVenus (VFP) was attached to the C-terminus of transmembrane domain. T-C6-VFP with C6 sequence replacing Alb and T-NO-VFP without Alb sequence incorporation was constructed using the same strategy. Tethered Alb domain constructs with sequence insertion of DI (residues 1-194), DII (residues 183-387), DIII (residues 381-585), DI-DII (residues 1-387), DII-DIII (residues 183-585), and DI-DIII (residues 1-194 and 381-585, respectively) were generated^47^ using PCR and then ligated into pMAX vector. Point mutations for hHv1 and T-Alb-VFP were introduced using QuikChange Site-Directed Mutagenesis Kit (Agilent). Primers used to generate point mutations and tethered Alb domain constructs are summarized in Supplementary Table 6. The sequences of all constructs were confirmed by DNA sequencing. Because hHv1 with V187C, L189C and L203C changes did not have expression in HEK293T cells, alanine mutations were studied at these three sites.

### Cell culture

HEK293T cells (RRID: CVCL_0063) were obtained from ATCC and used for heterologous expression. Cells were maintained in Dulbecco’s Modified Eagle Medium (DMEM) (ATCC) supplemented with 10% fetal bovine serum and 1% penicillin and streptomycin (Gibco) and incubated at 37 °C in a humidified atmosphere containing 5% CO_2_/95% air. Plasmids were transfected into cells using Lipofectamine 2000 (Life Technologies) according to the manufacturer’s instructions. Experiments were preformed 24–48 h post transfection.

### Proteins, peptides and reagents

C6 toxin (MH828728) was purchased as synthetic peptides from CSBio. Peptide toxin folding reactions were quenched by acidification and purified by reverse-phase HPLC, as before^5^. Peptides that were more than 95% pure were lyophilized and stored at −20 °C. The composition of the peptides was confirmed by mass spectral analysis. Peptides were dissolved in appropriate external solutions for whole-cell patch clamp recordings or physiological assays before use. Albumin from human serum (lyophilized powder, fatty acid free) was purchased from Sigma (A1887). Proteinase K (Lyophilized) was purchased from Promega (V3021) and the digestion was performed following the protocol supplied with the product. Purified Fab fragment of human IgG was purchased from GenWay Biotech (GWB-DD0665). Alb was labeled with 5,6-TAMRA-SE (5-(and-6-)-carboxytetramethyl-rhodamine succinimidyl ester) (Life Technologies) per manufacturer instructions. Thus, Alb was dissolved in 50 mM HEPES, 100 mM NaCl at pH 7.5 to a concentration of 10 mg/mL. 5,6-TAMRA-SE dye was dissolved in 500 µL DMSO. Alb and dye were mixed at a molar ratio of 1:10 for 1 h at room temperature on a rotating shaker. The labeled Alb was purified by HPLC over a 20–80% acetonitrile gradient and the corresponding single peak was collected. Samples were lyophilized and stored at −80 °C. Labeled protein was dissolved in appropriate external solution before use. All the reagents and chemicals were purchased from Sigma-Aldrich unless otherwise stated.

### Human sperm preparation

Ejaculates were obtained from healthy donors by masturbation after at least 48 h of sexual abstinence. Only semen samples that fulfilled the World Health Organization (WHO 2010) guidelines were selected for experiments. All semen donors gave written informed consent and the protocol for semen sample handling were approved by the Ethic Committee of the School of Medicine, National University of Cuyo and the Bioethics Committee at the Biotechnology Institute from the National Autonomous University of Mexico. After sample liquefaction (30 min at 37 °C), motile sperm were recovered after a swim-up separation for 1 h at 37 °C^5^.

### Human peripheral blood neutrophils purification

Human polymorphonuclear neutrophils were isolated from peripheral blood from healthy donors by Ficoll-Paque Plus (GE Healthcare) density-gradient centrifugation. Peripheral blood was obtained from Institute for Clinical and Translational Science of University of California Irvine and the protocol was approved by the Institutional Review Board of University of California Irvine. Donor population is composed of 50% female and 50% male with ages ranging from 23 to 62 years old. 20 mL blood was mixed with 3% dextran in PBS (Sigma-Aldrich) for 20 min in a 50 mL conical tube. The top clear layer containing leukocytes was collected and underlaid with 10 mL of Ficoll-Paque Plus. The cell suspension was centrifuged at 500 × *g* for 30 min at 20 °C to separate mononuclear cells from neutrophils and the remaining red blood cells. The overlying plasma and monocyte layers were aspirated, and the neutrophils and red blood cells pellet was re-suspended in Red Blood Cells Lysis Buffer (eBioscience), incubated for 10 min to lyse red blood cells. In total, 35 mL PBS was added to stop the lysis and the cell suspension was centrifuged at 300 × *g* for 5 min at 4 °C. Cell pellet was re-suspended in RPMI1640 (Gibco). An aliquot of neutrophils was mixed with Trypan blue (Gibco) and counted using a hemocytometer. Neutrophils isolated using this method were routinely found to be greater than 97% of the final cell preparation.

### Electrophysiology

Proton current from hHv1, CiHv1 and chimera channels were recorded in whole-cell mode using an Axopatch 200B amplifier. Stimulation and data collection were achieved with a Digidata1440A and pCLAMP 10 software (Molecular Devices). HEK293T cells expressing hHv1 channel variants were perfused with a nominally divalent cation-free external solution of 100 mM HEPES, 100 mM NaCl, 10 mM glucose at pH 7.5 or human tubular fluid medium (HTF) comprising 101.6 mM NaCl, 4.69 mM KCl, 0.2 mM MgSO_4_, 0.37 mM KH_2_PO_4_, 2.04 mM CaCl_2_, 25 mM NaHCO_3_, 2.78 mM Glucose, 0.33 mM Na pyruvate and 21.4 mM Na lactate at pH 7.2, unless otherwise noted. Pipettes with resistances between 3–5 MΩ were filled with 100 mM Bis-Tris buffer, 100 mM NaCl, and 10 mM glucose at pH 6.5 or 6.7. For EGTA recordings, 1 mM EGTA was added in the external solution with the supplement of 2 mM MgCl_2_. Sampling frequency was 10 kHz and was filtered at 1 kHz. Alb was applied in the external solution through a multichannel micro-perfusion system after currents monitored by test pulses to 0 mV for 1.5 s from a holding voltage of −60 mV, with 10 s interpulse intervals, were judged to be stable. Current-voltage relationships were evoked from a holding potential of −60 mV to test pulses from −60 mV to +60 mV for 1.5 s in 20 mV intervals every 10 s.

Proton currents in human neutrophils were recorded with an external solution of 100 mM HEPES, 130 mM NMDG, 10 mM glucose at pH 7.5. Pipettes with resistances between 10 and 15 MΩ were filled with 100 mM MES buffer, 130 mM NMDG, and 1 mM EGTA at pH 6.0. Current-voltage relationships were evoked from a holding potential of −60 mV to test pulses from −60 mV to +60 mV for 5 s in 20 mV intervals every 15 s. Current was assessed at the end of the test pulse.

The G-V relationships were determined as described by DeCoursey^66^, the reversal potential (V_rev_) is calculated with the equation V_rev_ = (I_end_- I_tail_)/(V_test_ – V_hold_), and were fit to the Boltzmann equation, G = G_max_/[1 + exp(-zF(V-V_1/2_)/RT)], where G_max_ is maximum conductance, V is the test potential, V_1/2_ is the voltage of half-maximal activation, z is the effective valence, T is the temperature, R is the gas constant, and F is the Faraday constant. Deactivation kinetics for hHv1 with and without Alb were determined by fitting traces with single exponential functions. Activation kinetics were fit with a single exponential having a delay. *k*_on_ and *k*_off_ were estimated from fits of the kinetics of toxin wash-in and wash-out and calculated using equations:

1 $${\tau }_{{\rm{on}}}={({k}_{{\rm{on}}}[{\rm{Tx}}]+{{k}}_{{\rm{off}}})}^{-1}$$

and

2 $${\tau }_{{\rm{off}}}={({k}_{{\rm{off}}})}^{-1}$$

A dose-response curve was determined by normalizing the current or the time constants of channel activation (τ_act_) to the values before the application of Alb, then plotting versus concentration of Alb. Dose-response curves were fitted with a Hill equation (Eq. (3)) in Origin 8.0.

3 $${\rm{r}}={[{\rm{Alb}}]}^{{\rm{h}}}/({{{\rm{EC}}}_{50}}^{{\rm{h}}}+{[{\rm{Alb}}]}^{{\rm{h}}})$$

where r is the rate of hHv1 current increasing with Alb at equilibrium, [Alb] is the concentration of Alb, and h is the Hill coefficient. The equilibrium affinity (*K*_*d*_) of Alb for hHv1 binding was estimated similarly by Hill equation, using the rate of hHv1 τ_act_ increasing and assuming Hill coefficient is 1 (simple bimolecular interaction).

Perforated patch clamp was performed with nystatin at 150 µg/ml in the pipette solution. After seal formation, the resting membrane potential of cells expressing hHv1 channels was measured in current-clamp configuration after attainment of whole-cell configuration with 10 mM HEPES, 136 mM KCl, 1 mM MgCl2, 2 mM K_2_ATP, 5 mM EGTA, pH 7.2 in the pipette and the bath solution described in the section Live cell FRET microscopy.

*Xenopus laevis* oocytes were injected with cRNA encoding hHv1 and proton current was measured using two-electrode voltage clamp (TEVC) three days thereafter. Recording solution for hHv1 was 60 mM NaCl, 1 mM MgCl_2_, 2 mM CaCl_2_, 120 mM HEPES, pH 7.3. Before hHv1 recording, we injected oocytes with 50 nL of 1 M HEPES (pH = 7.3) to minimize pH changes due to proton efflux. This results in ~100 mM HEPES in the cytosol. For recording the potassium currents, *Xenopus laevis* oocytes were injected with cRNA encoding hKv2.1 or h_S3-S4_Kv2.1. Recording solution was composed of 50 mM KCl, 50 mM NaCl, 1 mM MgCl2, 0.3 mM CaCl2, 10 mM HEPES, pH 7.5. Recordings were performed with constant gravity flow of solution at 2 mL/min yielding chamber exchange in ~5 s. Currents were recorded 1–2 days after cRNA injection using an Oocyte clamp amplifier OC-725C (Warner Instruments, Hamden, CT), and electrodes filled with 3 M KCl with resistance of 0.3 – 1 MΩ. Data were filtered at 1 kHz and digitized at 20 kHz using pCLAMP 10 and assessed with Clampfit 10 and Origin 8.

Procedures of human sperm electrophysiology were approved by the Bioethics Committee at the Biotechnology Institute from the National Autonomous University of Mexico. Motile sperm were recovered after a swim-up separation for 1 h in modified Krebs–Ringer bicarbonate medium under non-capacitation conditions (without BSA and Ca^2+^) at 37 °C in a humidified atmosphere of 5% CO_2_-95% air. Spermatozoa were stored in physiological solution comprising 135 mM NaCl, 5 mM KCl, 1 mM MgSO_4_, 2 mM CaCl_2_, 5 mM glucose, 1 mM sodium pyruvate, 10 mM lactose and 20 mM HEPES, pH 7.4 until used in electrophysiological recordings. Whole-cell patch clamp was used to record proton currents sealing at the cytoplasmic droplet from mature human spermatozoa plated on poly-lysine coated coverslips. Pipettes (20–30 MΩ) were filled with 135 mM N-methyl-D-glucamine (NMDG), 5 mM ethylene glycol tetraacetic acid (EGTA), and 100 mM MES adjusted to pH 6.0 with methanesulfonic acid (CH_3_SO_3_H). Seals between the patch pipette and the sperm cytoplasmic droplet were formed in physiological solution. After transition into whole-cell mode, the bath solution was changed to one that was free of added divalent cations comprised of 130 mM NMDG, 1 mM EDTA and 100 mM HEPES, pH 7.4 with CH_3_SO_3_H to measure dose-dependent activation of proton currents by Alb (Fig. 1a, b) and the G-V shift after application of 800 μM Alb (Fig. c). To determine the absolute effect of Alb on the G-V shift of sperm proton currents, we used the same bath solution without 1 mM EGTA (Supplementary Fig. 19 and Supplementary Table 1). CatSper currents were recorded in divalent cation-free solutions that contained 150 mM sodium gluconate, 2 mM Na_2_EDTA, 2 mM EGTA, 20 mM HEPES, pH 7.4, and the pipette solution contained 135 mM Cs-MeSO_3_, 5 mM CsCl, 5 mM Na-ATP, 10 mM EGTA, 20 mM HEPES, pH 7.0. For CatSper current recordings we used a conventional voltage-ramp protocol from −80 mV to +80 mV lasting 750 ms from a holding potential of 0 mV. Pulse protocol application and data acquisition were performed with a patch-clamp amplifier (Axopatch 200, Molecular Devices) and using the pCLAMP10 software (Molecular Devices). Data were sampled at 2–5 kHz and filtered at 1 kHz, and were analyzed with Clampfit 10 (Molecular Devices) and SigmaPlot 9.0 (Systat Software Inc.). Data were calculated and plotted as the mean ± standard error of the mean (SEM). All electrophysiological recordings were performed at 23 °C.

### Sperm intracellular pH measurements

After sperm sample liquefaction (30 min at 37 °C), highly motile sperm were recovered by swim-up separation for 1 h in HTF (see above), at 37 °C in an atmosphere of 5% CO_2_-95% air. Non-capacitated sperm were diluted to 10^7^ sperm/mL with modified HTF (mHTF: 4 mM NaHCO_3_ and 21 mM HEPES were used to replace 25 mM NaHCO_3_ in HTF) and loaded with BCECF (2 µM, cell-permeant, dual-excitation ratiometric fluorescent pH probe) in the dark at 37 °C for 30 min. Fluorescence intensity was measured with an Aminco Bowman II spectrofluorometer (λ_Ex1_ = 508, λ_Ex2_ = 450, λ_Em_ = 535). Non-capacitated sperm were allowed to stabilize until there was no change, or a small continuous change, in the fluorescent signal. Then Alb or Fab were added and changes in fluorescence were recorded for 400 s at a frequency of 0.5 Hz. High concentrations of Alb likely to cause quenching of fluorescent dyes, so 75 μM was the highest concentration tested. For the C6 group, non-capacitated sperm were incubated with 20 μM C6 for 1 h before the experiment. Conversion of the BCECF Ratio (508/450 nm) to pH value was performed using a calibration curve adjusted with a conventional pH electrode as previous described^28^. Statistical analyses were performed using the Dunnett Test.

### Sperm intracellular Ca^2+^ measurements

Non-capacitated sperm were diluted to 10^7^ sperm/mL with mHTF and loaded with Fluo3-AM (2 µM, cell-permeant fluorescent Ca^2+^ probe) in the dark at 37 °C for 30 min. Non-capacitated sperm were allowed to stabilize before 75 μM Alb was added and the changes in fluorescence intensity were measured (λ_Ex_ = 505 and λ_Em_ = 525) for 950 s at a frequency of 0.5 Hz. The increase in [Ca^2+^]_i_ was triggered by adding 15 μM progesterone. Raw intensity values were normalized as [(F/F0)-1], where F is fluorescence intensity at time t and F0 is the mean of F taken during the control period. In some studies, 10 μM ionomycin was applied after progesterone to study the ionophore-mediated rise in [Ca^2+^]_i_ and demonstrate that Alb did not alter dye loading; in this case normalization was to ionomycin induced peak [Ca^2+^]_i_. Statistical analyses were performed using the Dunnett Test.

### Sperm acrosome reaction measurements

Sperm suspensions were diluted to 10^7^ sperm/mL with HTF and incubated 3–4 h at 37 °C in an atmosphere of 5% CO_2_-95% air in presence of 5 mg/mL BSA to promote capacitation, or in absence of BSA for non-capacitating conditions. The effect of different concentrations of Alb, in the presence or absence of 20 μM C6 were tested. Spermatozoa were incubated for 30 min at 37 °C with 15 μM progesterone, spotted on slides, air-dried and stained with FITC-coupled *Pisum sativum* agglutinin (FITC-PSA, 25 μg/mL in PBS) for 40 min at room temperature. The presence of an intact acrosome was assessed in at least 200 cells per condition using an upright Nikon Optiphot II microscope equipped with epifluorescence optics. Statistical analyses were performed using Dunnett Test.

### Neutrophil ROS measurement

Human neutrophils were isolated from peripheral blood and re-suspended in Hank’s Balanced Salt Solution (HBSS) comprising 138 mM NaCl, 5.4 mM KCl, 0.34 mM Na_2_HPO_4_, 0.44 mM KH_2_PO_4_, 1.3 mM CaCl_2_, 0.5 mM MgCl_2_, 0.4 mM MgSO_4_, 4.2 mM NaHCO_3_, 5.5 mM glucose, and 20 mM HEPES, pH 7.2, and dispensed into white 96-Well Immuno Plates (2 × 10^5^ cells/well). Neutrophils were incubated with 500 μM Luminol (Sigma) and different concentrations of Alb for 30 min at 37 °C. For the C6 group, neutrophils were pre-incubated with 20 μM C6 for 30 min before the incubation with Luminol and Alb. After incubation, neutrophils were stimulated with fMLF and the chemiluminescence was measured immediately every 1 min for 60 min using Fluoroskan FL (ThermoFisher) equipped with internal software SkanIt 2.6.

### Neutrophil elastase measurement

Human neutrophils were washed and re-suspended in HBSS. Elastase release from neutrophils was evaluated using Elastase Substrate I, Colorimetric, AAPV-pNA (Millipore). All experiments were performed in polypropylene microcentrifuge tubes. Following the 30 min incubation with Alb or control protein Fab at 37 °C neutrophils (6.5 x l0^5^ cells per tube) were subjected to stimulation using 1 µM fMLF. All cells were incubated for another 5 min and subsequently centrifuged at 400 × *g* for 5 min. The cell free supernatant was added to individual microplate wells to achieve a total reaction volume of 200 µL per well and an AAPV-pNA concentration of 0.4 mM. Reactions were performed at 37 °C for 1 h, following which absorbance was measured at 405 nm. An extinction coefficient of 8.8 × l0^3^ cm/M was used to calculate the units of elastase released. This number was then divided by the total neutrophil elastase content as determined from the Triton X-100 incubated neutrophils yielding the percentage of total elastase release for each group.

### Two color smTIRF and photobleaching

HEK293T cells were seeded on glass bottom dishes (Chemglass Life Science) and transfected with hHv1-G199L-TFP. The surface density of channel molecules was kept less than 200 in a 10 × 10 µm field to minimize the overlapping of multiple channels within a diffraction-limited spot. TAMRA-Alb was added in 100 mM HEPES, 90 mM NaCl, 10 mM KCl, 0.5 mM CaCl_2_, 1.2 mM MgCl_2_, and 10 mM glucose, pH 7.5 to the dishes and incubate 30 min for reaching binding equilibrium. Cells were extensively washed to remove nonspecifically bound TAMRA-Alb before recording. Single protein molecules or complexes at the surface of live HEK293T cells were identified using TIRF microscope as described^5^. The critical angle for TIRF was adjusted using a CellTIRF illuminator (Olympus) and a high numerical aperture apochromat objective (150×, 1.45 NA) mounted on an automated fluorescence microscope controlled by Metamorph 7 software (Molecular Devices). Metamorph was used to simultaneously illuminate both fluorophores at a critical angle such that only 100 nm deep was illuminated. TAMRA was excited with the 561 nm laser line and TFP was excited with a 445 nm laser line. Emitted light signals were split with a 520 nm dichroic mirror mounted in a DualView adapter (Photometrics), which allows each wavelength to be directed to one half of a back-illuminated EM-CCD. Stoichiometry was assessed by simultaneous photobleaching with continual excitation. Data were captured as movies of 100–370 frames acquired at 1 Hz.

Data were analyzed as previously described^5^. When TAMRA was with TFP in the same cell, the data for each fluorophore were saved as separate stacks and processed in an identical manner. The Manders’ coefficient of colocalization (MMC) between fluorophores was determined by unbiased intensity correlation analysis using the Coloc2 plugin in ImageJ (Windows version) to confirm overlap of the two molecules. Fluorescence measured from each region was plotted versus time to determine the number of bleaching steps at each point. Statistical analyses to calculate estimated confidence with which stoichiometry could be inferred from the observed data and θ, the probability of successfully observing each possible photobleaching event, were performed in R Studio, based on methods developed by Hines^67^. The densities of colocalized and single fluorescent spots were determined following thresholding and watershed separation in ImageJ. Then the particle number was counted in separate regions for 3–5 cells per group using the Analyze particles plugin.

Single-molecule photobleaching events are missed in practical application to biological systems because of fluorophore prebleaching, the quantum efficiency of the fluorophore, and the time resolution of smTIRF system; in our studies, the error is estimated to be less than 10% as previously demonstrated^5,46^.

### Live cell FRET microscopy

Donor-decay time-course was studied as before^45^, using an Olympus inverted epi-fluorescence microscope. HEK293T cells were seeded on glass bottom dishes (Chemglass Life Science) and transfected with hHv1-TFP and T-Alb-VFP variants. Cells were recorded in a solution comprising 100 mM HEPES, 90 mM NaCl, 10 mM KCl, 0.5 mM CaCl_2_, 1.2 mM MgCl_2_, and 10 mM glucose, pH 7.5. Resting membrane potential (RMP) was altered by isotonic replacement of extracellular NaCl with KCl. For EGTA studies, 1 mM was added to the 10 mM KCl bath solution. TFP was excited at 445 nm and the emission collected through a 470–500 nm bandpass filter, VFP was excited at 514 nm and the emission collected through a 525–575 nm filter. Images were captured using a scientific camera controlled by Metamorph 7 software (Molecular Devices) and were analyzed with ImageJ^5,45^.

### Protein-peptide in silico docking

Unguided docking was performed using the amino acid sequence of the S3-S4 loop peptide of hHv1 (F190 to L200) and the crystal structure of Alb (PDB: 1BM0), to predict potential binding sites, using HPEPDOCK web server^48^.

### Molecular dynamics simulations

The initial structure of the dimeric hHv1 (only the transmembrane region: residues G90 to I218) at resting state was adopted from our previous study where the model was generated and refined by molecular dynamics (MD) simulations using spectroscopic constraints^43^. The hHv1 modeling structure was embedded in an explicit POPC lipid bilayer using the program VMD 1.93^68^, while the crystal structure of Alb (PDB 1BM0; residues S5 to A582) was placed in the extracellular side with its DII domain and the two potential binding sites facing the S3-S4 loops of hHv1, fully solvated in a 100 mM KCl solution. The final system contained ~185,000 atoms and was electrically neutral. The H193 on the subunit A of hHv1 was modeled in a protonated state to form hydrogen bonding interactions with E188 and E292 of Alb. Of note, the two glutamate residues could be the potential hydrogen bond donors as well.

The system was initially minimized for 5000 steps, followed by 10 ns equilibration with positional restraints (0.5 kcal/mol/Å^2^) being applied to the backbone of the whole Alb and the transmembrane helices of hHv1 to relax with the lipids. Then, the positional restraints were removed except the intracellular half of the transmembrane helicesS4 of hHv1 (residues R205 to I218), which were used to maintain the dimeric interface of hHv1 as its cytosolic coiled-coil structure was excluded from the model to minimize the size of the system. The distance of center of mass between the side-chain heavy atoms of hHv1-H193 (subunit A) and Alb-H288, and hHv1-H193 (subunit B) and Alb-H338 was gradually decreased (approaching to 5 Å) in 10 ns to pull Alb toward hHv1 using the Colvars module^69^. Meanwhile, the S1-S2 loops of the dimeric hHv1 were given a distance restraint for optimized Alb contact with hHv1. After that, 150 ns simulations were performed with different combinations of distance restraints between hHv1-H193 and their potential partners on Alb (Supplementary Table 4) to explore the interacting networks at the two binding sites and other contact regions of the complex where spontaneously and transiently formed hydrogen bonds between hHv1 and Alb had been found during the simulations. We used the distance restraints without angle restraints to determine if the enforced interaction was favorable. If the restrained interaction was favorable, a correct hydrogen-bonding angle formed automatically (Supplementary Fig. 14).

During the simulations, we found that E294 and E297 on Alb were close to I202 of hHv1, a helix turn upward the first gating charge R205, making E294 and E297 the probable negative countercharges activating the outward movement of the S4 helices of the dimeric hHv1. Thus, we generated and simulated another system of the Alb and hHv1 complex using the “up” state hHv1 (residues G90 to K221) from our previous study^43^. The simulation protocol was similar to the resting state model system with a total simulation time of 150 ns.

The MD simulations were carried out in the periodic boundary conditions with a time step of 2 fs using the NAMD 2.14 software program^49^. The CHARMM36 parameter set^70^ was used for proteins, lipids and ions, and the TIP3P model for water. The temperature and pressure were constrained at 300 K and 1 atm, respectively, using the Langevin dynamics and the Nose–Hoover Langevin piston method^71^. The long-range electrostatic force was calculated with the particle-mesh Ewald method^72^, and the short-range electrostatic and van der Waals interactions were smoothly switched off at 10−12 Å.

The evaluate the stability of the binding interactions of the two proteins, the two systems were further subjected to microsecond scale long simulations with ANTON2^50^, a special-purpose computer for long time scale MD simulations (Supplementary Fig. 15, 16, 17). The simulations were performed with 16 pairs of distance restraints, as shown in Supplementary Table 4, (derived from the MD simulations with NAMD) for 1.0 µs. These pairwise interactions were selected based on the examination of our initial MD simulation results with NAMD and our functional data. After that, another 0.5 µs run was performed only with the distance restraints being replaced by positional restraints on the backbone atoms of binding site residues (site 1: H288, E188, E292, R160, E153, and site 2: Y334, F374, R337, H338) of Alb and the backbone and C_β_, C_γ_ atoms of hHv1-H193. In all these ANTON2 simulations, positional restraints were applied on the alpha carbon atoms of the intracellular part of the transmembrane helices of hHv1 with the positional restraints on the backbone atoms of the intracellular part of S4 and the center-of-mass of the backbone atoms of the first two domains of Alb. The force filed, temperature, pressure, and time step were the same as those used in the MD simulations with NAMD. The temperature and pressure were constrained using the Nose–Hoover thermostat and the semi-isotropic MTK barostat^71^. PCA analysis was performed using the PyEMMA and Prody packages.

### Statistics

Statistical analyses were performed using the Dunnett Test, *P < 0.05, **P < 0.01, ***P < 0.001. Data are presented, where indicated as the mean ± standard error of the mean (SEM). The number of replicates for each study are described in the legends.

### Reporting summary

Further information on research design is available in the Nature Research Reporting Summary linked to this article.

## Supplementary information

Supplementary Information

Reporting Summary

## Data Availability

The data that support this study are available form the corresponding author upon reasonable request. The crystal structure of Alb that was used for molecular dynamics simulation is from Protein Data Bank entry 1BM0. The EPR-derived structural model of hHv1 and the computational model of the Alb-hHv1 complex are available from the corresponding author upon request. Source data is provided with this paper. Source data are provided with this paper.

## Source data

Source Data
