# Supplementary material for: Direct activation of the proton channel by albumin leads to human sperm capacitation and sustained release of inflammatory mediators by neutrophils
Source: Nat Commun. 2021 Jun 22;12:3855. doi: 10.1038/s41467-021-24145-1 (PMC8219737; doi:10.1038/s41467-021-24145-1)
Supplement: Supplementary file 1 — Supplementary Information [file 41467_2021_24145_MOESM1_ESM.pdf]

Direct activation of the proton channel by albumin leads to human sperm capacitation and sustained release of inflammatory mediators by neutrophils

Ruiming Zhao[1,6], Hui Dai[1,6], Rodolfo J. Arias[2], Gerardo A. De Blas[2,3], Gerardo Orta[4], Martín A. Pavarotti[2], Rong Shen[5], Eduardo Perozo[5], Luis S. Mayorga[2], Alberto Darszon[4] and Steve A. N. Goldstein[1*]

Supplementary information

Supplementary figure 1. Alb induces intracellular alkalization and enhances progesterone induced Ca^{2+} influx

Supplementary figure 2. Alb does not activate CatSper in human sperm

Supplementary figure 3. Alb activates neutrophils proton currents and increases fMLF-stimulated ROS production by human neutrophils

Supplementary figure 4. Alb activates hHv1-T29A

Supplementary figure 5. Physiological concentration and voltage dependent activation of Alb on hHv1

Supplementary figure 6. Human protein Fab or Proteinase K digested Alb does not potentiate hHv1 current

Supplementary figure 7. Alb does not activate other voltage-gated channels that were studied

Supplementary figure 8. Alb activates hHv1 expressed in *Xenopus* oocytes

Supplementary figure 9. Alb activation on Hv1Sper

Supplementary figure 10. Alb activates hHv1 in human tubal fluid

Supplementary figure 11. The S3-S4 loop of hHv1 confers Alb activation on to hKv2.1

Supplementary figure 12. ΔHv1 monomeric channels appear to be insensitive, or weakly activated, by Alb

Supplementary figure 13. *In silico* prediction of interaction between Alb and S3-S4 loop of hHv1

Supplementary figure 14. Molecular dynamics simulation system of the Alb-hHv1 complex

Supplementary figure 15. Structural stability of Alb and the Alb-hHv1 complex.

Supplementary figure 16. Principal component analysis of global and intra-molecular conformational changes of Alb during the simulations.

Supplementary figure 17. The distance distributions of two atoms or centers of mass of two groups of atoms from representative pairs of residues.

Supplementary figure 18. Cholesterol saturated Alb activates hHv1 like untreated Alb, and Alb activates hHv1-H140A but not hHv1-H193A channels

Supplementary figure 19. Alb activation of sperm proton currents without EDTA

Supplementary figure 20. EGTA activation of hHv1

Supplementary figure 21. Alb dose not activate hHv1-R205N, but activates hHv1-R211S channels normally

Supplementary table 1. Parameters for Alb activation of hHv1 in HEK293T cells, human sperm and neutrophils

Supplementary table 2. Stoichiometry of hHv1-G199L-TFP channels

Supplementary table 3. Parameters of single molecule photobleaching with hHv1-G199L TFP and TAMRA-Alb by smTIRF

Supplementary table 4. Distance restraints between pairs of residues used in the molecular dynamics simulations.

Supplementary table 5. Impact of EDTA and EGTA on the voltage-dependence of hHv1 activation ($V_{1/2}$) with and without Alb in sperm and HEK293T cells.

Supplementary table 6. Primers used in this study to generate hHv1 point mutations, tethered Alb domain constructs and Alb point mutations.

Supplementary figure 1. Alb induces intracellular alkalization and enhances progesterone-induced Ca^{2+} influx

Mature human spermatozoa were collected and studied using spectrofluorometry. pH_i and $[Ca^{2+}]_i$ were determined in non-capacitated sperm as described in Methods using BCECF and Flour-3, respectively. Values are the mean ± SEM. Reagent included 15 μM progesterone (Pg), 75 μM Alb or bovine serum albumin (BSA), the Fab fragment of human IgG (Fab) at 20 μM, C6 at 20 μM, the CatSper channel blocker NNC at 1 μM, the Ca^{2+} ionophore ionomycin (iono) at 10 μM, Statistical analyses were performed using the Dunnett Test or Student t-test, $*P < 0.05$, $**P < 0.01$, $***P < 0.001$. Source data are provided in the Source Data file.

a, pH_i changes were measured with BCECF and converted to ΔpH as described in Methods. Alb increased the speed of cytoplasmic alkalization in a concentration dependent manner (grey circles) whereas Fab did not (black circles, P=0.0001; one way ANOVA, Dunnett Test). C6 inhibited intracellular alkalization induced by Alb (red circles, P=0.0001; one way ANOVA, Dunnett Test), n = 3-13 independent experiments.

b, The increase in $[Ca^{2+}]_i$ triggered by Pg was measured in the absence of Alb (left) and after adding Alb (middle). The fluorescence increase stimulated by Pg in presence of Alb was inhibited by C6 (right).

c, The presence of Alb (grey bar, P=0.01; one way ANOVA, Dunnett Test) increased peak $[Ca^{2+}]_i$ stimulated by Pg 2.4-fold over baseline and this increase was inhibited by C6 (red bar) or NNC (blue bar), n = 3-5 independent experiments.

d, Representative recordings of $[Ca^{2+}]_i$ without (left) or with (right) BSA in arbitrary fluorescence units (arb. units). BSA enhanced $[Ca^{2+}]_i$ influx induced by Pg. The absolute rise in au on subsequent addition of iono was similar with BSA (0.31 ± 0.05 au) and without BSA (0.39 ± 0.03 au) indicating similar dye loading in the two conditions.

e, $[Ca^{2+}]_i$ changes normalized to peak arb. units on addition of iono.

f, Five independent samples of human sperm with BSA (right, grey bar, P=0.04; two-sided Student t-test) showed a 2.2-fold increase in peak $[Ca^{2+}]_i$ compared to control (left, white bar) here calculated as ΔPg/Δiono, where ΔPg and Δiono are the change in peak $[Ca^{2+}]_i$ induced by Pg and iono, respectively, n = 5 independent experiments.

g, pH_i changes were measured as described in panel a. BSA increased the speed of cytoplasmic alkalization and BSA pre-saturated and applied with a 10-fold molar excess cholesterol had a similar effect (P=0.8; one way ANOVA, Dunnett Test) on the rate of pH_i increase as did untreated BSA alone, n= 3-6 independent experiments.

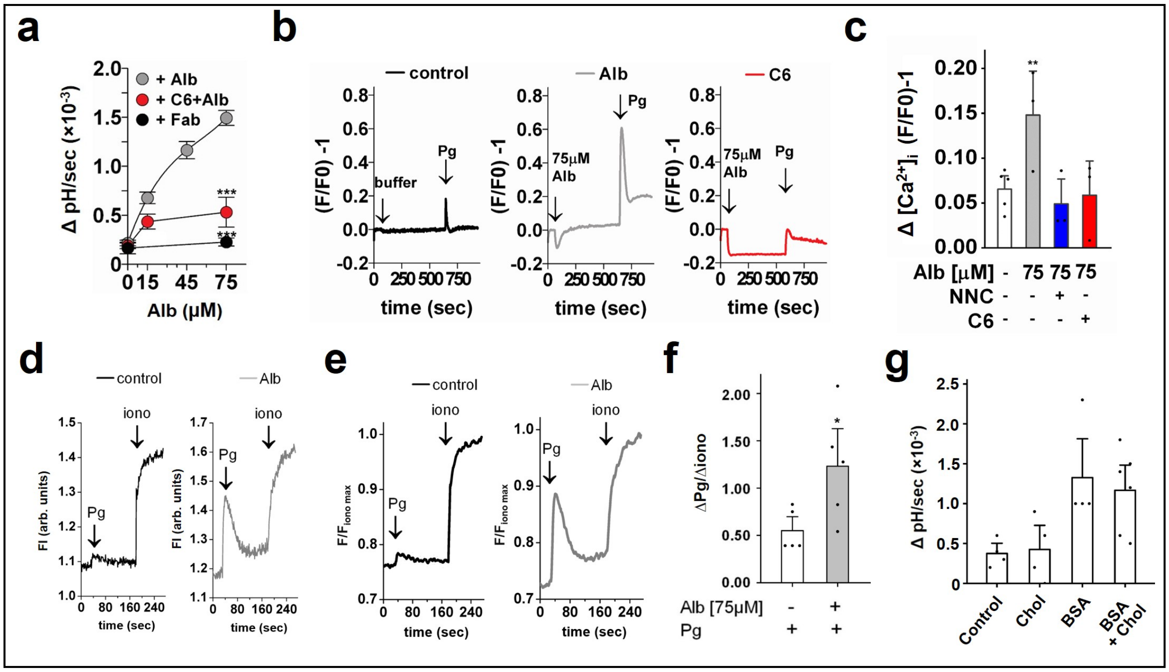

Supplementary figure 2. Alb does not activate CatSper in human sperm

A typical voltage-ramp protocol was used for recording monovalent CatSper current in whole-cell voltage-clamp experiments with non-capacitated human sperm, as described in Methods. Source data are provided in the Source Data file.

a, The representative monovalent CatSper current trace in the absence (black trace) and presence (red trace) of 80 µM Alb, or in presence of 10 mM NH_4Cl (blue trace) are shown.

b, Relative current amplitudes measured at +80 and -80 mV from experiments as shown in panel a. Values are mean ± SEM, n = 5 cells.

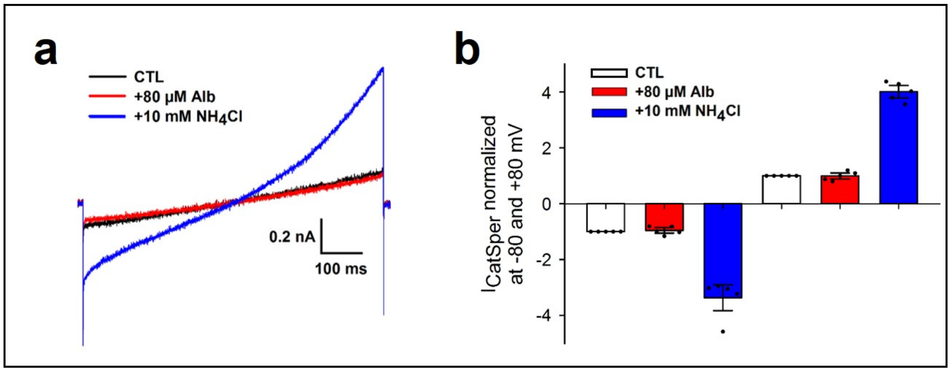

Supplementary figure 3. Alb activates proton currents and increases fMLF-stimulated ROS production by human neutrophils

As described in Methods, human neutrophils were isolated from peripheral blood of healthy volunteers and recorded by whole-cell patch clamp or measured using a microplate luminometer for ROS production. Values are mean ± SEM. Source data are provided in the Source Data file.

a, Proton currents via hHv1 in human neutrophils were studied as described in Fig. 2a with a ~30-fold proton gradient (pH_i = 6.0 and pH_o = 7.5). G-V for neutrophils proton currents in the absence (black squares) or presence of 450 μM Alb (black circles). The proton conductance in neutrophils showed a -35 ± 3 mV shift after exposure to 450 μM Alb. Curves are fitting to the Boltzmann equation as described in Methods, n = 3 cells.

b, The ROS production was measured as described in Fig. 2b. fMLF alone (1 μM) stimulated ROS production by human neutrophils which could be inhibited by 20 μM C6 (red bar), while 450 μM Alb alone did not increase ROS production comparing to unstimulated human neutrophils (Control), n = 3 independent experiments.

c, Normalized ROS production by human neutrophils without (black circles) or with the incubation of 450 μM Alb (grey circles) stimulated with an increasing concentration of fMLF equivalent to the Fig. 2c. Values of the total ROS production were normalized such that the ROS production stimulated by 0.01 nM and 10 μM fMLF correspond to *0* and *1*, respectively. Some error bars are smaller than symbols, n = 3 independent experiments.

d, Effect of 10 μM fMLF alone or in combination with 450 μM Alb on ROS production by human neutrophils. Incubation with Alb enhanced and sustained the ROS production by human neutrophils.

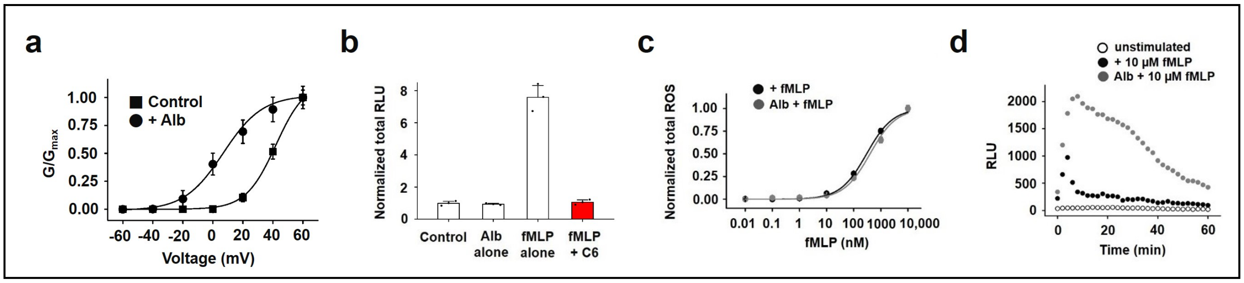

Supplementary figure 4. Alb activates hHv1-T29A.

hHv1-T29A channels, carrying change in PKC-δ phosphorylation site at the N terminus of hHv1 were expressed in HEK293T cells and studied using whole-cell voltage clamp with 1.5 s pulses from a holding voltage of -60 mV with 10 s interval and a 10-fold proton gradient (pH_i = 6.5 and pH_o = 7.5). 75 μM Alb (red trace) was applied after control pulses (black trace). Alb-activation increases hHv1-T29A currents of ~3-fold at 0 mV, n = 3 cells.

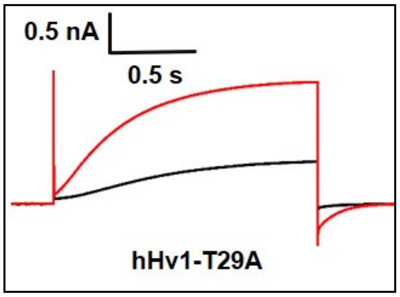

Supplementary figure 5. Voltage dependent activation of hHv1 by Alb at 500 μM, the concentration in the female reproductive tract

hHv1 channels expressed in HEK293T cells were studied using whole-cell voltage clamp with 1.5 s pulses from a holding voltage of -60 mV with 10 s interval and a 10-fold proton gradient (pH_i = 6.5 and pH_o = 7.5). Values are mean ± SEM. Some error bars are smaller than symbols. Source data are provided in the Source Data file.

a, Representative proton current traces for hHv1 channels before (top), and in the presence of 500 μM Alb (bottom) with steps of 20 mV increments from −60 mV to +60 mV.

b, Conductance-voltage relationships (G-V) for hHv1 in the absence (black squares) or presence of 500 μM Alb (black circles). hHv1 channels showed a 45 ± 4 mV shift after exposure to 500 μM Alb (Supplementary table 1). Curves are fitted to a Boltzmann equation as described in Methods, n = 6 cells.

c, Dose-response relationships for Alb on increasing of the activation time constants (τ_{act}) of hHv1 at depolarizing voltages of 0 mV and +60 mV. The activation of proton currents at 0 mV and +60 mV were fitted with a single exponential function to determine the extent of Alb activation on τ_{act}. Values of the ratio of τ_{act} in the presence and absence of Alb were normalized such that the ratio of τ_{act} in the presence of 1 μM Alb and 800 μM Alb values correspond to *0* and *1*, respectively. The equilibrium affinity (K_d) of Alb for hHv1 channels activation at 0 mV and +60 mV was estimated from the fit to Hill relationship as 84.5 ± 9.8 μM with a Hill coefficient of 1.08 ± 0.13, and 54.6 ± 6.7 μM with a Hill coefficient of 1.19 ± 0.14, respectively, n = 6 cells.

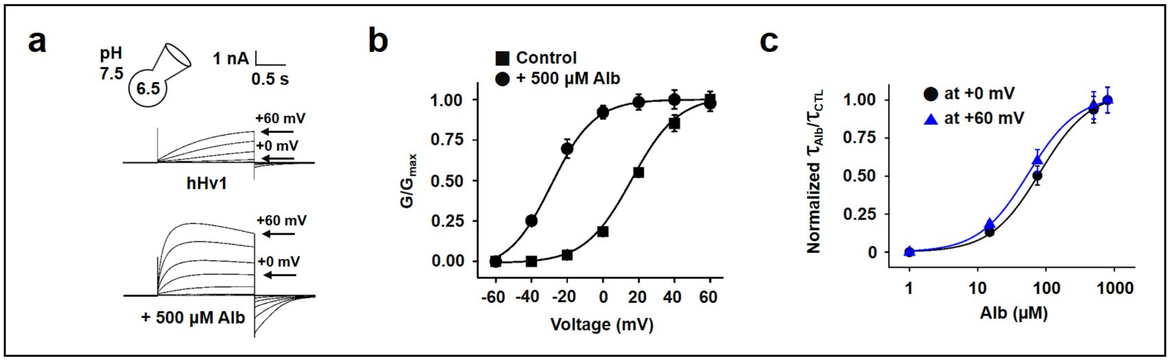

Supplementary figure 6. Human protein Fab or Proteinase K-digested Alb do not potentiate hHv1 current

hHv1 channels expressed in HEK293T cells were studied using whole-cell voltage clamp with 1.5 s pulses to 0 mV from a holding voltage of -60 mV with 10 s interval and a 10-fold proton gradient (pH_i = 6.5 and pH_o = 7.5).

a, 800 µM Fab (red trace) was applied after control pulses (black trace) and shows no activation of the proton current.

b, Alb was incubated and digested with proteinase K at a concentration of 800 µM, as described in Methods. Alb after digestion (red trace) was applied after control pulses (black trace) and shows no activation of the proton current.

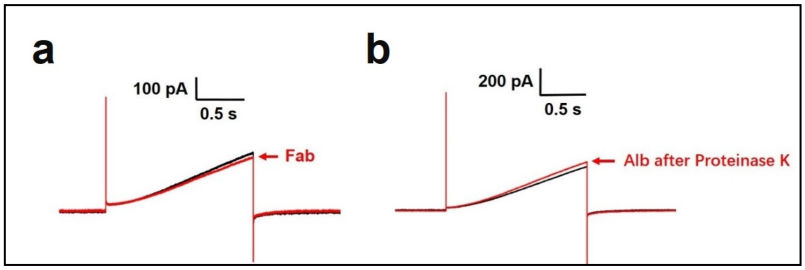

Supplementary figure 7. Alb does not activate other voltage-gated channels that were studied

a-d, Voltage gated potassium channels were expressed in HEK293T cells, and studied using 300 ms (hKv1.3, hKv2.1, hKv1.5) or 1.5 s (hI_{ks}) pulses to 0 mV from a holding voltage of -80 mV with 10 s interval. Bath solution comprises 130 mM NaCl, 4 mM KCl, 1.2 mM $MgCl_2$, 2 mM $CaCl_2$, and 10 mM HEPES; pH was adjusted to 7.4 with NaOH. Electrodes were filled with a solution containing 130 mM KCl, 1 mM $MgCl_2$, 5 mM EGTA, 5 mM K_2ATP, and 10 mM HEPES; pH was adjusted to 7.4 with KOH. 75 µM Alb (red trace) was applied after control pulses and shows no activation comparing to control current before application (black trace).

e, hNav1.5 was expressed in HEK293T cells, and studied using 20 ms pulses to 0 mV from a holding voltage of -100 mV with 10 s interval. Bath solution comprises 130 mM NaCl, 4 mM CsCl, 2 mM $CaCl_2$, 1.2 mM $MgCl_2$, 5.5 mM glucose, and 10 mM HEPES, pH 7.4 with NaOH. Electrodes were filled with a solution containing 60 mM CsCl, 80 mM CsF, 1 mM $CaCl_2$, 1 mM $MgCl_2$, 5 mM Na_2ATP, 10 mM EGTA and 10 mM HEPES, pH 7.4 with CsOH. 75 µM Alb (red trace) was applied after control pulses and shows no activation comparing to control current before application (black trace).

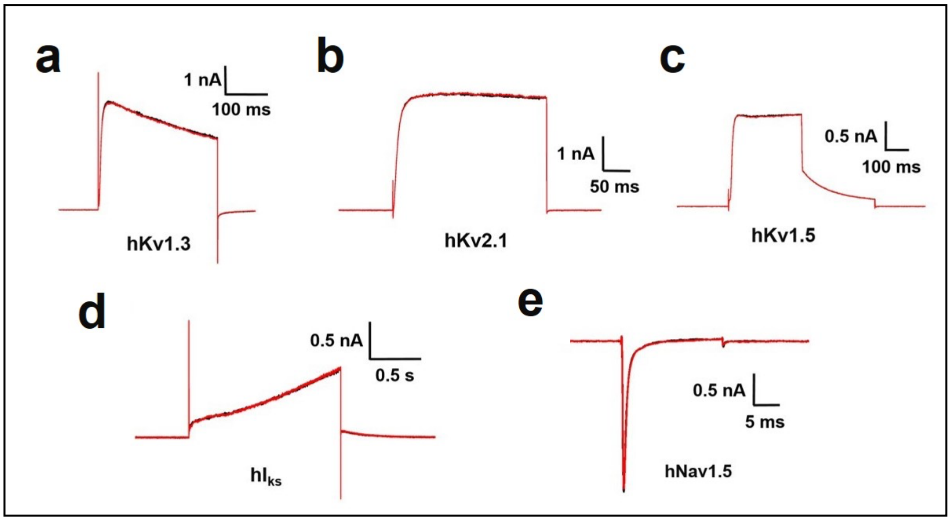

Supplementary figure 8. Alb activates hHv1 expressed in *Xenopus* oocytes

hHv1 channels were expressed in *Xenopus* oocytes and studied by two-electrode voltage clamp (TEVC) with a holding voltage of -60 mV and 1.5 s steps to 100 mV every 10 s in the absence of proton gradient ($pH_i = pH_o = 7.3$) as described in Methods. 75 µM Alb (red trace) was applied after control pulses (black trace) and increased hHv1 currents ~1.8-fold (n = 3).

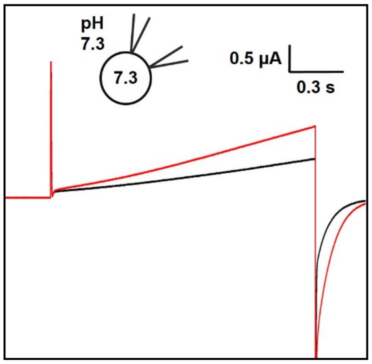

Supplementary figure 9. Alb activation on Hv1Sper

Hv1Sper was constructed by truncating 67 residues at the N-terminus of hHv1 (residues from A2 to R68). Hv1Sper channels expressed in HEK293T cells were studied using whole-cell voltage clamp with 1.5 s pulses from a holding voltage of -80 mV with 10 s interval and a 10-fold proton gradient ($pH_i = 6.5$ and $pH_o = 7.5$). Values are mean ± SEM, n = 3 cells for each condition. Some error bars are smaller than symbols. Curves are fitted to the Boltzmann equation. Source data are provided in the Source Data file.

a, Representative proton current traces for Hv1Sper channels before (left), and in the presence of 500 µM Alb (right) with steps of 20 mV increments from −80 mV to +60 mV.

b, G-V for Hv1Sper in the absence (black squares) or presence of 500 µM Alb (black circles). Hv1Sper channels showed a 15 ± 2 mV shift after exposure to 500 µM Alb (Supplementary table 1).

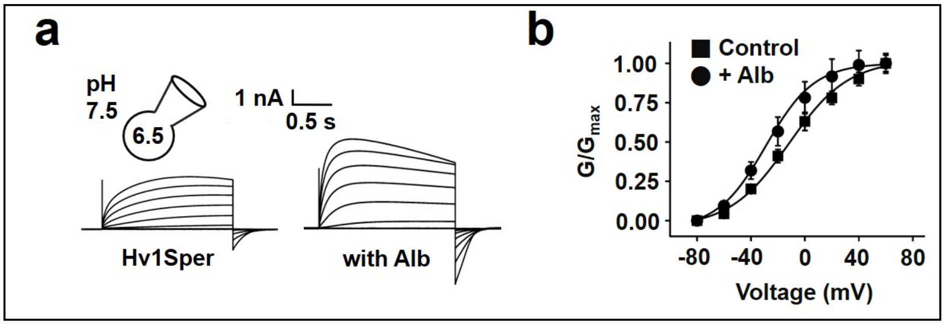

Supplementary figure 10. Alb activates hHv1 in human tubal fluid
hHv1 channels expressed in HEK293T cells were studied using whole-cell voltage clamp with 1.5 s pulses from a holding voltage of -60 mV with 10 s interval and a 3-fold proton gradient (pH_i = 6.7 and pH_o = 7.2) in human tubal fluid (HTF) medium. Values are mean ± SEM, n = 3 cells for each condition. Some error bars are smaller than symbols. Source data are provided in the Source Data file.

a, Representative proton current traces for hHv1 channels in HTF medium before (left), and in the presence of 500 μM Alb (right), with steps of 20 mV increments from −60 mV to +60 mV.

b, G-V for hHv1 in HTF medium in the absence (black squares) or presence of 500 μM Alb (black circles). hHv1 channels showed a 40 ± 4 mV shift in $V_{1/2}$ after exposure to 500 μM Alb (Supplementary table 1). The voltage at which hHv1 channels start to open ($V_{threshold}$) shifted from ~0 mV to -30 mV with Alb application. Curves are fit to a Boltzmann equation.

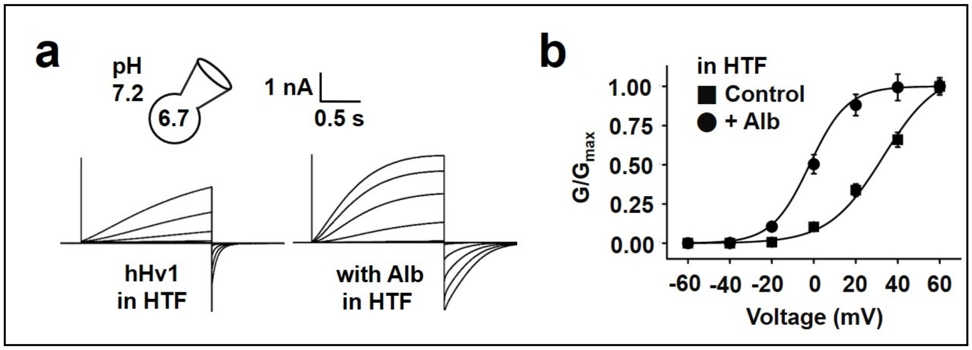

Supplementary figure 11. The S3-S4 loop of hHv1 confers Alb activation to hKv2.1
The $h_{S3\text{-}S4}$Kv2.1 chimera channel was constructed as described in Methods. hKv2.1 and $h_{S3\text{-}S4}$Kv2.1 were expressed in *Xenopus* oocytes and studied by TEVC with a holding voltage of -80 mV and 0.5 s steps to -20 mV every 10 s.

a, Sequence alignment of hKv2.1 (cyan), hHv1 (black) and $h_{S3\text{-}S4}$Kv2.1 chimera transplanting the S3-S4 loop of hHv1 into hKv2.1.

b, Representative current trace for hKv2.1 and $h_{S3\text{-}S4}$Kv2.1 without (black) or with 500 μM Alb application (red). hKv2.1 was insensitive to 500 μM Alb, while the same concentration of Alb increased K^+ current through $h_{S3\text{-}S4}$Kv2.1 ~1.9-fold (n = 3).

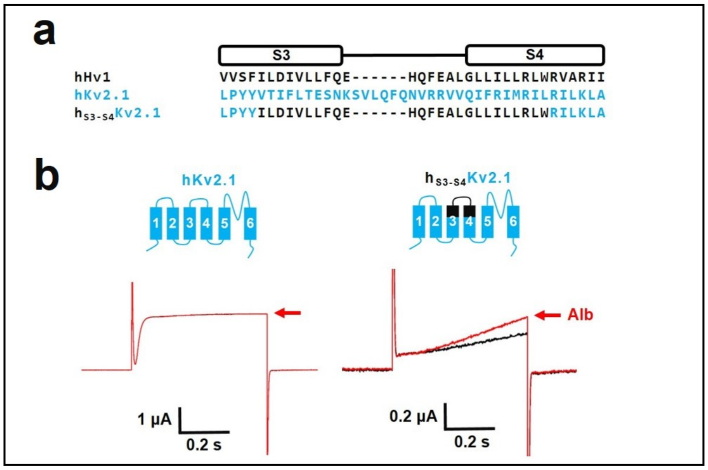

Supplementary figure 12. ΔHv1 monomeric channels appear to be insensitive, or weakly activated, by Alb

ΔHv1 channels were expressed in HEK293T cells and studied by whole-cell patch clamp to assess activation parameters using a holding voltage of −60 mV, 1.5 s test pulses, and a 10 s interpulse interval, pH_i = 6.5, pH_o = 7.5. Values are mean ± SEM. Some error bars are smaller than symbols. Source data are provided in the Source Data file.

a, Representative proton current traces for ΔHv1 channels before (left), and in the presence of 800 μM Alb (right) with steps of 20 mV from −60 mV to 40 mV. Fitting the activation and deactivation of proton currents at 0 mV to a single exponential function gave time constants τ_{act} of 350 ± 42 ms and 152 ± 19 ms, τ_{tail} of 33 ± 5 ms and 60 ± 9 ms without and with 800 μM Alb, respectively. The observed activation is suspected to be due to a subpopulation of dimeric channels that forms with the truncated subunits.

b, Dose-response relationships for Alb activation of ΔHv1. EC_{50} of Alb for ΔHv1 channels was estimated from the fit to Hill relationship as 1,904 ± 155 μM with a Hill coefficient of 1.05 ± 0.06. The maximal Alb (800 μM) potentiated currents amplitude on WT hHv1 (Fig. 3d) was set as the maximal effect for fitting, n = 3-8 cells for each condition.

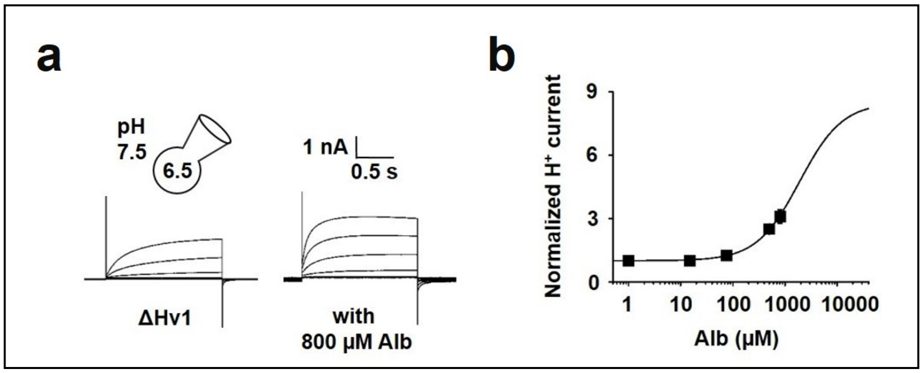

Supplementary figure 13. ***In silico*** **prediction of interaction between Alb and S3-S4 loop of hHv1**
A computational protein-peptide docking web server (HPEPDOCK){Zhou, 2018 #110} was used to predict the binding configurations of a minimal 11-residue binding epitope (F190 to L200) within the S3-S4 loop of hHv1 on the Alb crystal structure (PDB: 1BM0). HPEPDOCK performed the protein-peptide docking through a hierarchical algorithm. Top 20 low energy models were favorably clustered in two binding sites, with 25% S3-S4 loop peptides clustered in Site 1, while 70% in Site 2 (three binding poses in Site 1 and two binding poses in Site 2 are shown). The distance between two predicated binding sites is ~27 Å, matching the distance (~26 Å) between two individual hHv1 subunits of one dimeric channel.

a, Docking result for two low energy poses of S3-S4 loop peptide (cyan and lime) with Alb, showing binding Site 1 on DI (grey) and DII (orange) of Alb is one hot spot for hHv1 channel interaction.

b, Close-up view of interaction interface between S3-S4 loop peptide and Alb in Site 1 in a docking model. The hHv1-H193 (cyan) could be a partner to Alb-E188, Alb-H288 and Alb-E292.

c, Docking result for three low energy poses of S3-S4 loop peptide (blue, green, and red) with Alb, showing binding Site 2 on DII (orange) of Alb is the other hot spot for hHv1 channel interaction.

d, Close-up view of interaction interface between S3-S4 loop peptide and Alb in Site 2 in a docking model. The hHv1-H193 (blue) could be a partner to Alb-Y334, Alb-R337, Alb-H338, Alb-D340, Alb-F374 and Alb-V381.

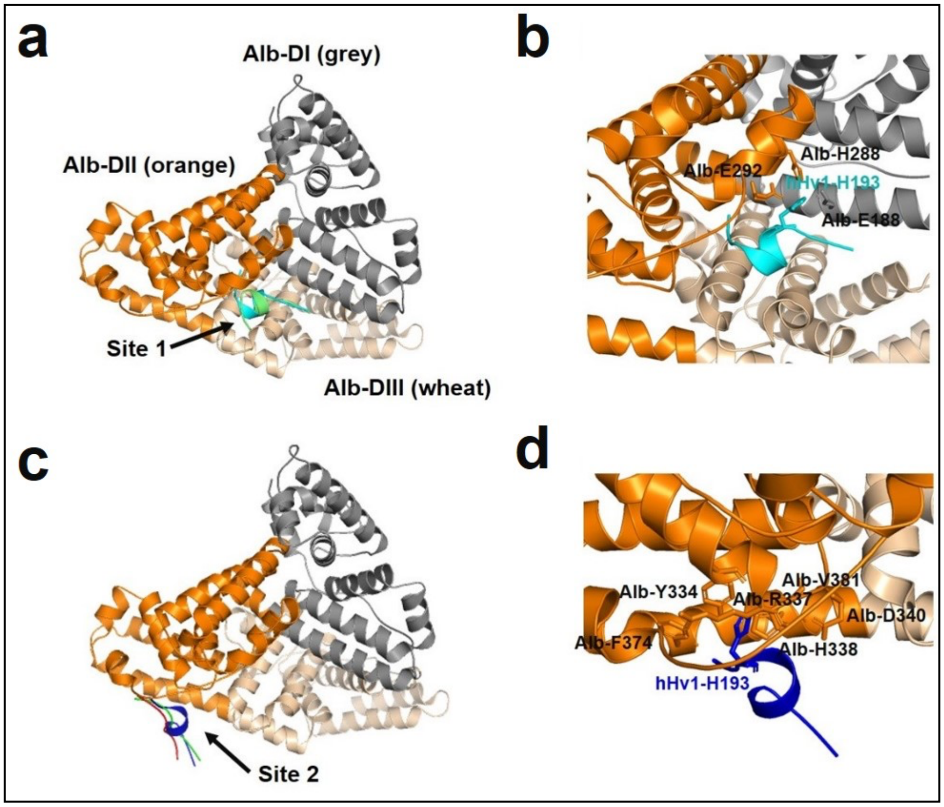

Supplementary figure 14. Molecular dynamics simulation system of the Alb-hHv1 complex

Starting configuration of Alb and hHv1 for all-atom MD simulations. The Alb, hHv1 and water were shown in a surface representation, and the lipid molecules and ions in spheres, respectively. The Alb was orientated with its DII (orange) facing the extracellular side of the dimeric hHv1 (cyan for subunit A, blue for subunit B). Some lipid molecules were removed for the sake of clarity.

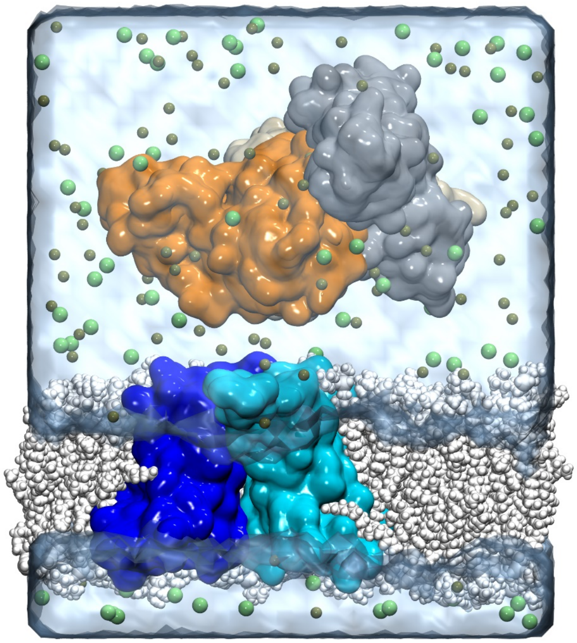

Restraints were persistently used during long-term stimulation in order to maintain the Alb-hHv1 complex (Methods). The reasons were (1) the large thermal fluctuation of Alb. The size of Alb is very large, comprising of 578 residues in the model, compared with its binding partner the S3-S4 loop of hHv1 which is about 10 amino acids long. (2) Only the H193 residue on hHv1 was actively involved in the residue-residue interaction networks with Alb at channel "down" state. (3) The steric clash between the third domain of Alb and the S1-S2 loop of hHv1. The root-mean-square deviation (RMSD) analysis and principle component analysis (PCA) of the 1.5 μs ANTON2 trajectories showed that there is a dramatic steric clash between Alb-DIII and the S1-S2 loop of hHv1 (Supplementary Fig. 15 and 16). This unfavorable interaction will push Alb away from hHv1, which is in good agreement with our FRET microscopy results showing that the Alb domain variant containing only the first two domains (Alb-DI-DII) has higher binding affinity to hHv1 than the WT Alb (Fig. 5h). (4) the lack of the atomic structure of the dimeric hHv1. We used the homology model of hHv1 from our previous study to build and refine the Alb-hHv1 complex. The deviation of the hHv1 homology model itself may bias our simulation results of the Alb-hHv1 complex.

Due to the large size of the system (~185,000 atoms), we cannot simulate the complete binding/unbinding process to further evaluate the binding conformation. However, our RMSD and PCA results based on the multi-microseconds long restrained simulations showed that the global binding conformation between Alb and hHv1 is stable considering the big size of Alb, the flexibility of the S3-S4 loop of hHv1 at the binding interface, and only a few distance/positional restraints surrounding hHv1-H193 have being applied. In addition, by analyzing the last 0.5 μs ANTON2 trajectories, we found that the important hydrogen binding and π-π stacking interactions shown in Fig. 6 still exist (Supplementary Fig. 17), considering the corresponding distance restraints had already been replaced by a few positional restraints during the simulations. It implies that these critical residue-residue interactions are favorable in the proposed model of the complex, or in the binding state of the two proteins.

Supplementary figure 15. Structural stability of Alb and the Alb-hHv1 complex.
Time series of the backbone RMSDs of Alb and its three domains Alb-DI, Alb-DII, Alb-DIII in reference to the corresponding initial (red), final (blue) and crystal (black) structures in the 1.5 μs ANTON2 simulation of the Alb-hHv1$_{down}$ system (**a**) and the Alb-hHv1$_{up}$ system (**b**). No initial structural alignment was performed in the RMSD calculations using the initial and final structures as reference to show the movement of Alb during the MD simulations. The vertical dashed line highlights the time boundary of two periods of the simulation. Top-view (left panel) and side-view (right panel) of the final structure of the Alb-hHv1$_{down}$ complex (**c**) and the Alb-hHv1$_{up}$ complex (**d**). The crystal structure of Alb was superimposed in transparent ribbon representation, showing the distortion of the Alb-DIII domain due to its steric clash with the S1-S2 loop of hHV1 (highlighted in the dashed box). The subunit A of hHv1 and the Alb-DI domain are not shown in the side-view for clarity.

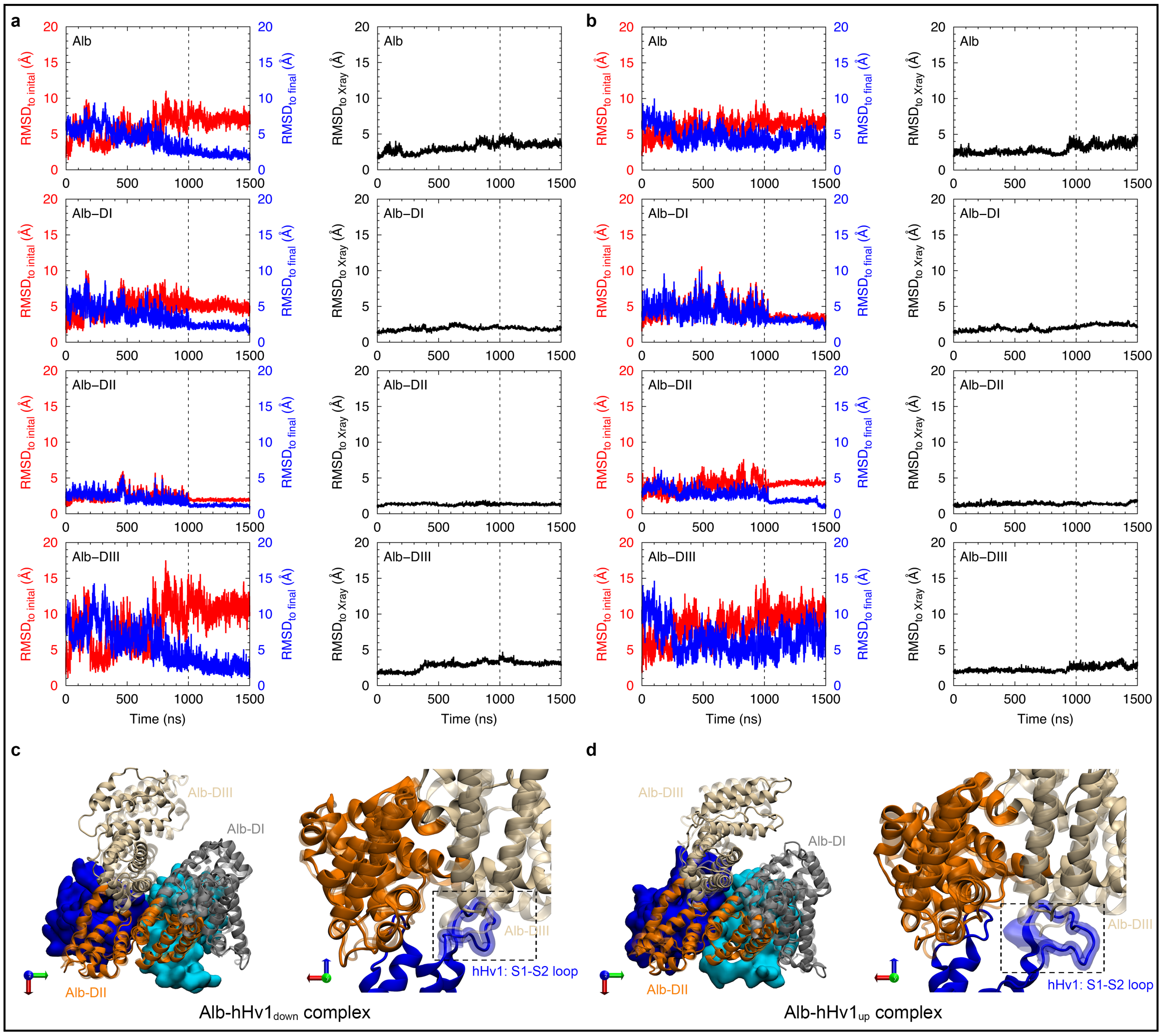

Supplementary figure 16. Principal component analysis of global and intra-molecular conformational changes of Alb during the simulations.

Principal component analysis (PCA) was performed on Cartesian coordinates of the Cα atoms of Alb generated in the 1.5 μs ANTON2 simulation of the Alb-hHv1$_{down}$ system (**a, b, c**) and the Alb-hHv1$_{up}$ system (**d, e, f**). Structural alignment based on the backbone atoms of the DII domain of the crystal structure was performed before the PCA analysis of the intra-molecular conformational changes of Alb (**b, c, e, f**), while no initial structural alignment was performed for the PCA analysis of the global conformational changes of Alb (**a, d**). The projections of all the conformations (*n* = 2084) onto the first two principal components, PC1 and PC2, are shown as heat maps (**a, b, d, e**). Projections of the initial, final and crystal structures onto PC1 and PC2 are depicted as scattered points. **c**, **f**, Comparison of the square displacement of each residue along PC1 and PC2.

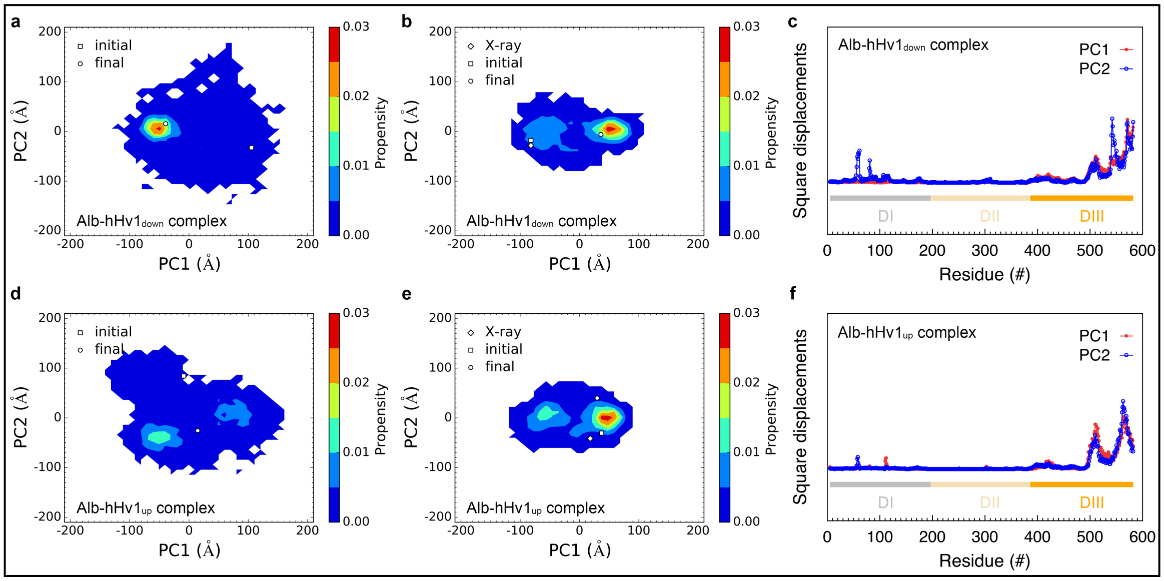

Supplementary figure 17. The distance distributions of two atoms or centers of mass of two groups of atoms from representative pairs of residues.

Snapshots from the last 500 ns ANTON2 trajectories of the Alb-hHv1$_{down}$ (**a**) and Alb-hHv1$_{up}$ (**b**) systems were used for the calculation (*n* = 2084). The number in the parentheses represents the hydrogen bond propensity between the two residues. A hydrogen bond is defined as the distance between the hydrogen donor and acceptor atoms is shorter than 3.5 Å and the angle between the donor, the hydrogen and the acceptor atoms is larger than 120°.

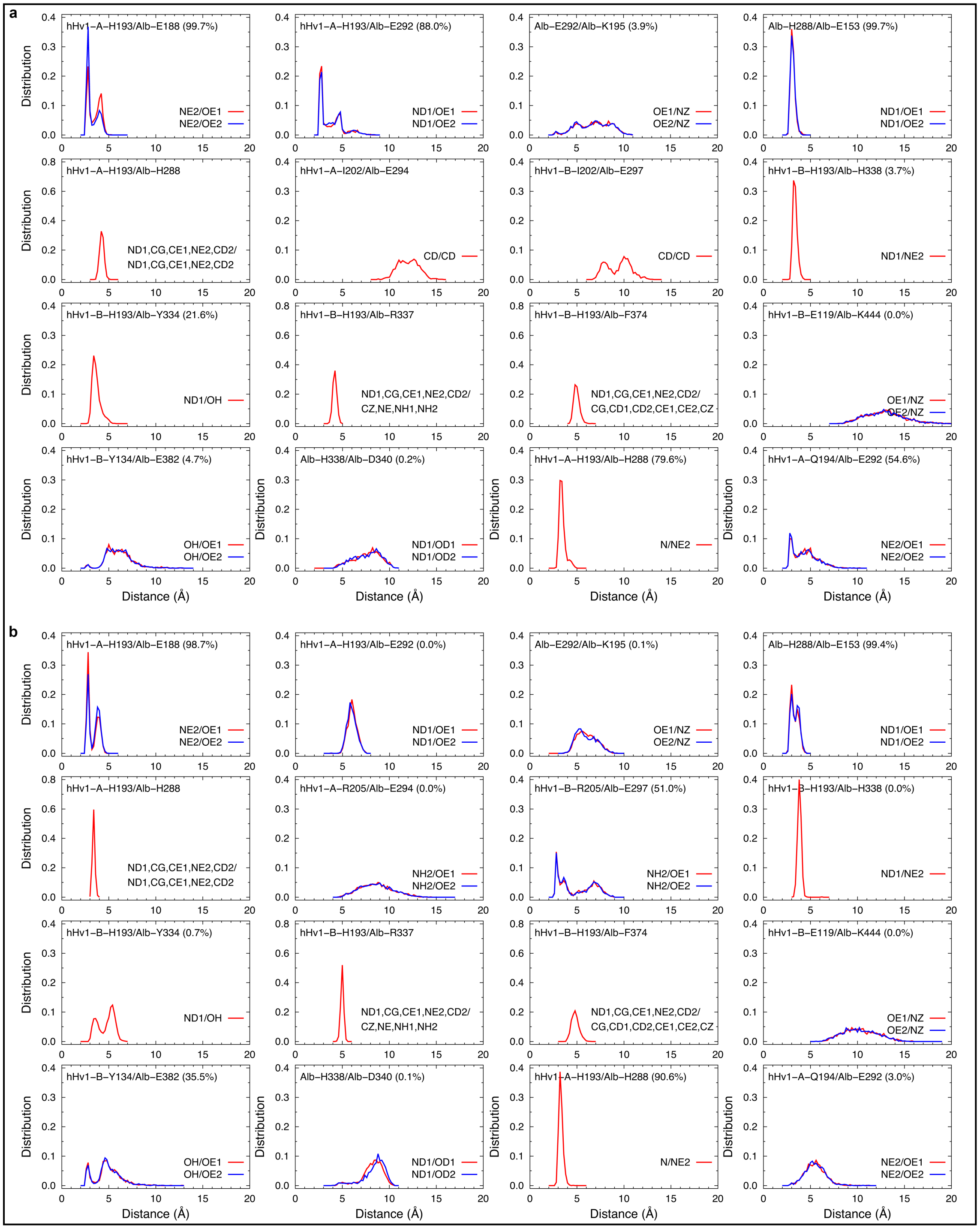

Supplementary figure 18. Cholesterol saturated Alb activates hHv1 like untreated Alb, and Alb activates hHv1-H140A but not hHv1-H193A channels

a, hHv1 channels were expressed in HEK293T cells, and studied using whole-cell voltage clamp with 1.5 s pulses to 0 mV from a holding voltage of -60 mV with 10 s intervals with a 10-fold proton gradient (pHi = 6.5 and pHo = 7.5) and divalent cation-free bath solution. 75 μM Alb that was presaturated with sodium cholesteryl sulfate (red trace) was applied after control pulses (black trace) and increased hHv1 currents ~8-fold (n = 3).

b-c, hHv1-H193A and hHv1-H140A channels, carrying changes in either the Zn^{2+} binding histidine residue in the S3-S4 loop or in the S1-S2 loop, respectively, were expressed in HEK293T cells, and studied using whole-cell voltage clamp with 1.5 s pulses to 0 mV from a holding voltage of -60 mV with 10 s interval. 75 μM Alb (red trace) was applied after control pulses (black trace). The H193A mutation disrupts activation so 75 μM Alb has no effect on hHv1-H193A (panel b), same as shown in Fig. 4c for hHv1-H193C; in contrast, mutation of the Zn^{2+} binding residue in the S1-S2 loop did not alter Alb-activation showing an increase in hHv1-H140A currents of ~6-fold (panel c) (n = 3).

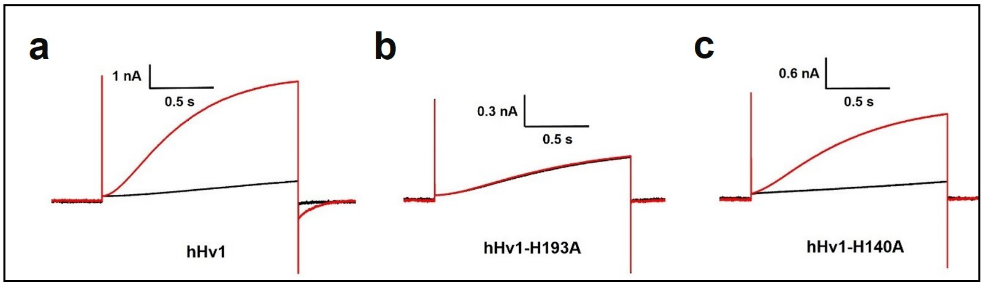

Supplementary figure 19. Alb activation of sperm proton currents without EDTA

As described in the Methods, mature human spermatozoa were collected and recorded by whole-cell patch clamp. G-V for sperm proton conductance showed a -53 ± 5 mV shift in $V_{1/2}$ after exposure to 500 μM Alb in EDTA free recording solution from 15 ± 2 mV to -38 ± 3 mV (Supplementary table 2). The $V_{threshold}$ shifted from ~-30 mV to -70 mV with Alb application (Supplementary table 1). Curves are fitting to the Boltzmann equation as described in Methods. Values are mean ± SEM, n = 5 cells. Source data are provided in the Source Data file.

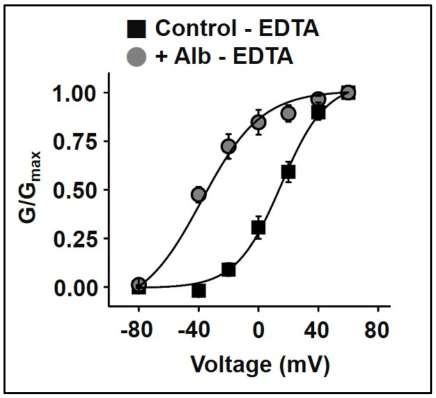

Supplementary figure 20. EGTA activates hHv1

hHv1 channels expressed in HEK293T cells were studied using whole-cell voltage clamp with 1.5 s pulses from a holding voltage of -60 mV with 10 s interval and a 10-fold proton gradient (pH_i = 6.5 and pH_o = 7.5) with addition of 2 mM $MgCl_2$ (Methods). Values are mean ± SEM, n = 3 cells for each condition. Some error bars are smaller than symbols. Source data are provided as a Source Data file.

a, Representative proton current traces for hHv1 channels before (left), and in the presence of 1 mM EGTA (right), with steps of 20 mV increments from −80 mV to +40 mV.

b, G-V for hHv1 in the absence (black squares) or presence of 1 mM EGTA (black circles). hHv1 channels showed a 40 ± 3 mV shift after exposure to 1 mM EGTA from 15 ± 1 mV to -25 ± 2 mV. Curves are fitting to the Boltzmann equation.

c, 500 μM Alb (red trace) was applied after control pulses after pre-activation with 1 mM EGTA (black trace) and shows attenuated activation of the proton current.

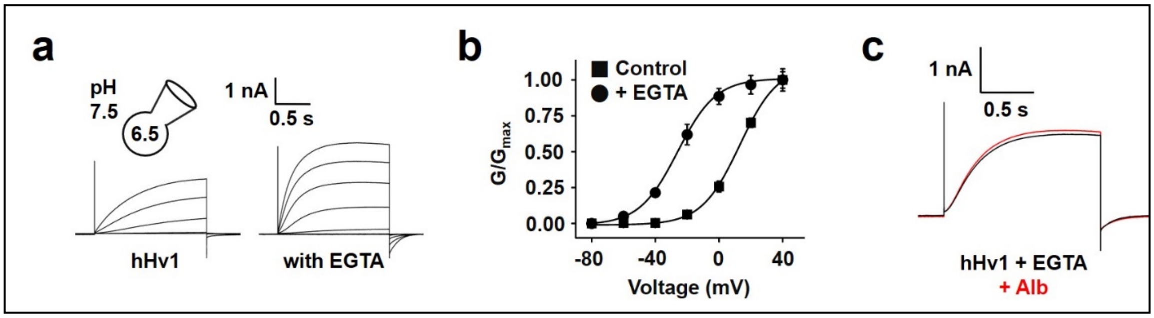

Supplementary figure 21. Alb does not activate hHv1-R205N, but does activate hHv1-R211S

hHv1-R205N and hHv1-R211S channels, carrying changes in the first and the third voltage sensing Arg residue in S4, respectively, were expressed in HEK293T cells, and studied using whole-cell voltage clamp with 1.5 s pulses to 0 mV (hHv1-R205N) or +40 mV (hHv1-R211S) from a holding voltage of -60 mV with 10 s interval with a 10-fold proton gradient (pHi = 6.5 and pHo = 7.5) and divalent cation-free bath solution. After control pulses (black trace), 75 μM Alb (red trace) was applied.

a, The R205 mutation fully eliminate activation by 75 μM Alb

b, The mutation of R211 did not alter Alb-activation showing an ~5-fold increase in current (n=3).

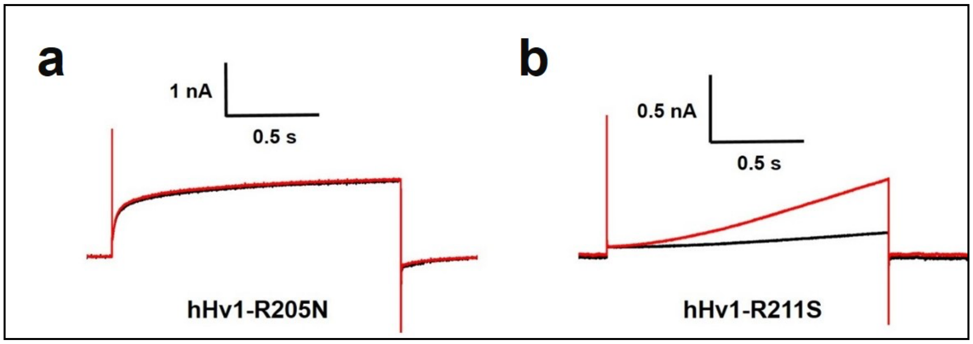

Supplementary table 1. Parameters for Alb activation of hHv1 in HEK293T cells, human sperm and neutrophils

Channel in HEK293	Without Alb			With 500 μM Alb					
	$V_{1/2}$, mV	τ activation, ms	τ deactivation, ms	$V_{1/2}$, mV	τ activation, ms	τ deactivation, ms	$V_{1/2}$ shift	I_{Alb}/I_{CTL}, 0 mV	EC_{50} (μM)
Condition: pH_i = 6.5 and pH_o = 7.5									
**hHv1**	15 ± 2	2620 ± 220	86.9 ± 8.4	-30 ± 2	99.4 ± 9.2	418 ± 47	45	8 ± 1	74.8 ± 8.7
**h_{S3-S4}CiHv1**	27 ± 4	4728 ± 936	39.1 ± 7.5	-16 ± 4	171 ± 32	196 ± 37	43	9 ± 1	63.3 ± 12.5
**hHv1-G199L**	18 ± 3	2553 ± 126	63.9 ± 6.1	-28 ± 3	72.1 ± 11.7	530 ± 84	46	8 ± 1	3.4 ± 0.5
**Hv1Sper**	-16 ± 2	369 ± 57	170 ± 38	-31 ± 4	87.3 ± 19.1	236 ± 31	15	1.6 ± 0.2	ND
				**With T-Alb-VFP**					
**hHv1**				3 ± 1	872 ± 163	175 ± 37	12	3.1 ± 0.3	
Condition: pH_i = 6.7 and pH_o = 7.2 (Human tubular fluid medium)									
**hHv1**	37 ± 3	11624 ± 652	79.8 ± 4.3	-3 ± 1	349 ± 27	461 ± 25	40	10 ± 1	ND
**Native Cells**	**Without Alb**			**With 500 μM Alb**					
Condition: pH_i = 6.0 and pH_o = 7.4									
**Sperm H^+ current**	15 ± 2	1846 ± 40	94 ± 12	-38 ± 3	217 ± 21	486 ± 63	53	7.6 ± 1.3	158 ± 16
	**Without Alb**			**With 450 μM Alb**					
Condition: pH_i = 6.0 and pH_o = 7.5									
**Neutrophil H^+ current**	42 ± 2	383 ± 65	111 ± 18	7 ± 1	63.8 ± 9.2	165 ± 25	35	2.5 ± 0.3	112 ± 9

Legend. WT hHv1, h_{S3-S4}CiHv1, hHv1-G199L and Hv1Sper were expressed in HEK293T cells and studied by whole-cell patch clamp, as described in Fig. 3 and Supplementary Fig. 10. Proton currents in human sperm (without EDTA in recording solution) and neutrophils were studied by whole-cell patch clamp, as described in Figs. 1, 2 and Methods. Half-maximal activation voltages ($V_{1/2}$) were determined by fits to a Boltzmann function, as described in Methods and Fig. 3b. τ for activation and deactivation were determined from single exponential fits, as described in Fig. 3a. ND, not determined. Data are mean ± SEM for 3-8 cells studied for each channel construct. Recording solutions are described in the Methods.

Supplementary table 2. Stoichiometry of hHv1-G199L-TFP channels

	**Subunits expressed**
	**hHv1-G199L-TFP**
**Determined stoichiometry**	2
**Particles studied**	86
**Particles with**	
**One step**	7
**Two steps**	77
**Three steps**	0
**Four steps**	2
**Confidence**	0.999
**θ**	0.96
**θ+ 1**	0.64

Legend. hHv1-G199L-TFP was expressed in HEK293T cells and studied by smTIRF (Methods). The number of photobleaching steps observed for TFP in each single fluorescent spot reports on the stoichiometry of channels. Like WT hHv1[5], hHv1-G199L-TFP channels are dimeric. Analysis was performed according to methods described by Hines[66]. The statistical confidence in the null hypotheses that hHv1-G199L-TFP forms dimers was assessed to be greater than 0.999. Prebleaching and variance in quantum efficiency reduce the probability of observing each possible bleaching event (θ). θ is calculated from the value of n and the distribution of the photobleaching data[66]. θ is decreased when the distribution is altered to estimate the possibility that higher numbers of missed bleaching steps, for example θ + 1, indicating that this stoichiometry is less likely.

Supplementary table 3. Parameters of single molecule photobleaching with hHv1-G199L-TFP and TAMRA-Alb by smTIRF

**Protein expressed/applied**	**hHv1-G199L-TFP + TAMRA-Alb**
**Determined stoichiometry**	2:1
**Manders' coefficient of colocalization**	0.50 ± 0.06
**Particles studied**	
**Total**	86
**Two TFP (dimeric channels)**	77
**Colocalized TFP and TAMRA**	39
**Dimeric hHv1 with one Alb**	37
**Dimeric hHv1 with two Alb**	2

Legend. hHv1-G199L-TFP was expressed in HEK293T cells and incubated with 3 μM TAMRA-Alb to reach equilibrium. Simultaneous, two-color, single particle photobleaching was studied by TIRF (Methods). Mean Manders' coefficient of colocalization were generated by using unbiased intensity correlation analysis as described in the Methods. The number of colocalized particles and photobleaching steps observed for TFP and TAMRA fluorophores in each single fluorescent spot were analyzed as previously described[5] and used for determining the binding stoichiometry of Alb with channels.

Supplementary table 4. Distance restraints between pairs of residues used in the molecular dynamics simulations.

Residue *i*	Residue *j*
hHv1-B-H193: ND1,CG,CE1,NE2,CD2	Alb-Y334: CG,CD1,CE1,CZ,OH,CD2,CE2
hHv1-B-H193: ND1,CG,CE1,NE2,CD2	Alb-R337: CZ, NH1, NE, NH2
hHv1-B-H193: ND1,CG,CE1,NE2,CD2	Alb-H338: ND1,CG,CE1,NE2,CD2
hHv1-B-H193: ND1,CG,CE1,NE2,CD2	Alb-F374: CG,CD1,CD2,CE1,CE2,CZ
hHv1-B-H193: CE1	Alb-F374: CG,CD1,CD2,CE1,CE2,CZ
hHv1-B-E119: OE2	Alb-K444: NZ
hHv1-B-D123: OD2	Alb-R445: NH2
hHv1-B-K125: NZ	Alb-E393: OE1
hHv1-B-K125: NZ	Alb-E396: OE1
hHv1-B-Y134: OH	Alb-E382: OE1
hHv1-B-E192: OE1	Alb-K378: NZ
hHv1-B-E193: CG	Alb-F374: CG
hHv1-B-H193: CE1	Alb-F374: CZ
hHv1-B-H193: ND1	Alb-Y334: OH
hHv1-B-H193: ND1	Alb-R337: NH1
hHv1-B-H193: ND1	Alb-H338: NE2
hHv1-B-H193: NE2	Alb-A306: N
hHv1-B-H193: NE2	Alb-Y334: OH
hHv1-B-H193: NE2	Alb-R337: NH1
hHv1-B-H193: NE2	Alb-H338: NE2
hHv1-B-I202: CD	Alb-E297: CD
hHv1-B-R205: NH2	Alb-E297: CD
hHv1-A-H193: ND1,CG,CE1,NE2,CD2	Alb-E188: OE1,OE2,CD
hHv1-A-H193: ND1,CG,CE1,NE2,CD2	Alb-H288: ND1,CG,CE1,NE2,CD2
hHv1-A-H193: ND1,CG,CE1,NE2,CD2	Alb-H288: CE1
hHv1-A-Y134: OH	Alb-E280: OE1
hHv1-A-Y141: OH	Alb-E280: OE1
hHv1-A-E192: OE1	Alb-R160: NH1
hHv1-A-H193: CD2	Alb-H288: NE2
hHv1-A-H193: CD2	Alb-H288: CG
hHv1-A-H193: CE1	Alb-H288: CE1
hHv1-A-H193: ND1	Alb-E188: OE1
hHv1-A-H193: ND1	Alb-E188: OE2
hHv1-A-H193: ND1	Alb-H288: CD2
hHv1-A-H193: ND1	Alb-H288: CG
hHv1-A-H193: ND1	Alb-H288: NE2
hHv1-A-H193: ND1	Alb-E292: OE1
hHv1-A-H193: ND1	Alb-E292: OE2
hHv1-A-H193: NE2	Alb-E188: OE1
hHv1-A-H193: NE2	Alb-E188: OE2
hHv1-A-H193: NE2	Alb-H288: CE1
hHv1-A-H193: NE2	Alb-H288: ND1
hHv1-A-H193: NE2	Alb-H288: NE2
hHv1-A-H193: NE2	Alb-E292: OE2
hHv1-A-H193: N	Alb-H288: NE2
hHv1-A-Q194: OE1	Alb-K195: NZ
hHv1-A-Q194: NE2	Alb-E292: OE1
hHv1-A-E196: OE2	Alb-K439: NZ
hHv1-A-I202: CD	Alb-E294: CD
hHv1-A-R205: NH2	Alb-E294: CD
Alb-E188: CD	Alb-R160: CZ
Alb-E188: OE1	Alb-R160: NH2
Alb-E188: OE2	Alb-R160: NH1
Alb-E188: OE2	Alb-R160: NH2
Alb-H288: ND1	Alb-E153: OE1
Alb-H288: ND1	Alb-E153: OE2
Alb-H288: NE2	Alb-R160: NH2
Alb-E292: CD	Alb-K195: NZ
Alb-E292: OE1	Alb-K195: NZ
Alb-H338: ND1	Alb-D340: OD1

Legend. The distance between two selected atoms or centers of mass of two groups of atoms was harmonically restrained with a force constant of 5 kcal/mol/Å^2 or less, centered at 3.5 Å for the hydrogen bond donor/accepter pairs and 4 or 5 Å for the rest. Different combinations of the restraints were applied during the refinement process. The restraints applied in the ANTON2 simulations were highlighted in red.

Supplementary table 5. Impact of EDTA and EGTA on the voltage-dependence of hHv1 activation ($V_{1/2}$) with and without Alb in sperm and HEK293T cells

Sperm H^+ current $V_{1/2}$ in mV (pH_i = 6.0, pH_o = 7.4)			
	**Before (Control)**	**After application**	**Shift**
**Alb (500 uM)**	15 ± 2	-38 ± 3	53
**EDTA (1 mM)**	15 ± 2	4 ± 1	11
**EGTA (1 mM)**	17 ± 3	-10 ± 4	27
**Alb (800 uM) + EDTA (1 mM)**	15 ± 2	-28 ± 2	43
**hHv1 in HEK293T cells, $V_{1/2}$ in mV (pH_i = 6.5, pH_o = 7.5)**			
	**Before (Control)**	**After application**	**Shift**
**Alb (500 uM)**	15 ± 2	-30 ± 2	45
**EDTA (1 mM)**	15 ± 2	-29 ± 3	44
**EGTA (1 mM)**	15 ± 1	-25 ± 2	40
**Alb (500 uM) + EDTA (1 mM)**	15 ± 2	-30 ± 3	45
**Alb (500 uM) + EGTA (1 mM)**	15 ± 1	-25 ± 3	40

Legend. Proton currents in human sperm were studied by whole-cell patch clamp, as described in Fig. 1, Supplementary Fig. 19 and Methods. hHv1 were expressed in HEK293T cells and studied by whole-cell patch clamp, as described in Fig. 3 and Supplementary Fig. 20. The $V_{1/2}$ of channel activation were determined by fits to a Boltzmann function, as described in Methods. Data are mean ± SEM for 3-8 cells studied for each condition. Recording solutions are described in the Methods.

Supplementary Table 6. Primers used in this study to generate hHv1 point mutations, tethered Alb domain constructs and Alb point mutations.

Construct	Forward primer	Reverse primer
hHv1-I183C	ACAGGAGGACAATGTCGAGACAGAATGAGACCACCACCACG	CGTGGTGGTGGTCTCATTCTGTCTCGACATTGTCCTCCTGT
hHv1-I184C	CTGGAACAGGAGGACAATGTCACAGATGAATGAGACCACCACCAC	GTGGTGGTGGTCTCATTCATCTGTGACATTGTCCTCCTGTTCCAG
hHv1-D185C	CTCCTGGAACAGGAGGACAATACAGAGGATGAATGAGACCACCAC	GTGGTGGTCTCATTCATCCTCTGTATTGTCCTCCTGTTCCAGGAG
hHv1-I186C	TCCTGGAACAGGAGGACACAGTCGAGGATGAATGAGAC	GTCTCATTCATCCTCGACTGTGTCCTCCTGTTCCAGGA
hHv1-V187A	GTGCTCCTGGAACAGGAGAGCAATGTCGAGGATGAATG	CATTCATCCTCGACATTGCTCTCCTGTTCCAGGAGCAC
hHv1-L188C	AAACTGGTGCTCCTGGAACAGACAGACAATGTCGAGGATGAATGA	TCATTCATCCTCGACATTGTCTGTCTGTTCCAGGAGCACCAGTTT
hHv1-L189A	CAAACTGGTGCTCCTGGAAAGCGAGGACAATGTCGAGGATG	CATCCTCGACATTGTCCTCGCTTTCCAGGAGCACCAGTTTG
hHv1-F190C	GTGCTCCTGGCACAGGAGGACAATGTCGAG	CTCGACATTGTCCTCCTGTGCCAGGAGCAC
hHv1-Q191C	GAGCCTCAAACTGGTGCTCGCAGAACAGGAGGACAATGTCG	CGACATTGTCCTCCTGTTCTGCGAGCACCAGTTTGAGGCTC
hHv1-E192C	CCAGAGCCTCAAACTGGTGGCACTGGAACAGGAGGACAATG	CATTGTCCTCCTGTTCCAGTGCCACCAGTTTGAGGCTCTGG
hHv1-H193C	CAGAGCCTCAAACTGGCACTCCTGGAACAGGAGG	CCTCCTGTTCCAGGAGTGCCAGTTTGAGGCTCTG
hHv1-Q194C	GGCCCAGAGCCTCAAAGCAGTGCTCCTGGAACAGG	CCTGTTCCAGGAGCACTGCTTTGAGGCTCTGGGCC
hHv1-F195C	CAGAGCCTCACACTGGTGCTCCTGGAACAG	CTGTTCCAGGAGCACCAGTGTGAGGCTCTG
hHv1-E196C	CAGGCCCAGAGCGCAAAACTGGTGCTCCTGGAACAGGA	TCCTGTTCCAGGAGCACCAGTTTTGCGCTCTGGGCCTG
hHv1-A197C	ATCAGCAGGCCCAGACACTCAAACTGGTGCTCC	GGAGCACCAGTTTGAGTGTCTGGGCCTGCTGAT
hHv1-L198C	AATCAGCAGGCCGCAAGCCTCAAACTGGTGCTCCTGGA	TCCAGGAGCACCAGTTTGAGGCTTGCGGCCTGCTGATT
hHv1-G199C	GCAGAATCAGCAGGCACAGAGCCTCAAACTG	CAGTTTGAGGCTCTGTGCCTGCTGATTCTGC
hHv1-G199L	CCGTAGCAGAATCAGCAGTAGCAGAGCCTCAAACTGGTG	CACCAGTTTGAGGCTCTGCTACTGCTGATTCTGCTACGG
hHv1-L200C	CAGCCGTAGCAGAATCAGGCAGCCCAGAGCCTCAAACTG	CAGTTTGAGGCTCTGGGCTGCCTGATTCTGCTACGGCTG
hHv1-L201C	CACAGCCGTAGCAGAATGCACAGGCCCAGAGCCTCAA	TTGAGGCTCTGGGCCTGTGCATTCTGCTACGGCTGTG

hHv1-I202C	CACAGCCGTAGCAGACACAGCAGGCCCAGAGC	GCTCTGGGCCTGCTGTGTCTGCTACGGCTGTG
hHv1-L203A	GCCACAGCCGTAGAGCAATCAGCAGGCCCAGAGCC	GGCTCTGGGCCTGCTGATTGCTCTACGGCTGTGGC
hHv1-L204C	CACCCGCCACAGCCGACACAGAATCAGCAGGCC	GGCCTGCTGATTCTGTGTCGGCTGTGGCGGGTG
hHv1-R205N	CCACCCGCCACAGATTTAGCAGAATCAGCAGGCCCA	TGGGCCTGCTGATTCTGCTAAATCTGTGGCGGGTGG
hHv1-R211S	TAATCCCATTGATGATGCTGGCCACCCGCCACAG	CTGTGGCGGGTGGCCAGCATCATCAATGGGATTA
T-Alb-DI-VFP	CAGTTGCTACGCGTGACGCCCACAAGAGCGAGGTGG	AACTTCCGCTAGCGGCGGAGCTGGCCTTGCCCTCATC
T-Alb-DII-VFP	CAGTTGCTACGCGTGACGAGCTGCGGGATGAGGGCAAG	GAACTTCCGCTAGCCAGGTTCTGGGGTTCCTCCACC
T-Alb-DIII-VFP	CAGTTGCTACGCGTGTGGAGGAACCCCAGAACCTGATC	GAACTTCCGCTAGCGAGTCCCAGAGCAACCTGAGAG
T-Alb-DI- DII-VFP	CAGTTGCTACGCGTGACGCCCACAAGAGCGAGGTGG	GAACTTCCGCTAGCCAGGTTCTGGGGTTCCTCCACC
T-Alb-DII-DIII-VFP	CAGTTGCTACGCGTGACGAGCTGCGGGATGAGGGCAAG	GAACTTCCGCTAGCGAGTCCCAGAGCAACCTGAGAG
T-Alb-E184A	CTCATCCCGCAGCGCGTCCAGCTTAGG	CCTAAGCTGGACGCGCTGCGGGATGAG
T-Alb-E188A	CTGGCCTTGCCCGCATCCCGCAGCT	AGCTGCGGGATGCGGGCAAGGCCAG
T-Alb-E285A	GATACAGTGGCTCTTTGCCAGCAGGGGCTTCTC	GAGAAGCCCCTGCTGGCAAAGAGCCACTGTATC
T-Alb-H288A	CACCTCGGCGATACAGGCGCTCTTTTCCAGCAGG	CCTGCTGGAAAAGAGCGCCTGTATCGCCGAGGTG
T-Alb-E292A	CGTCGTTCTCCACCGCGGCGATACAGTGG	CCACTGTATCGCCGCGGTGGAGAACGACG
T-Alb-E311A	CACGTCCTTGCTCGCCACGAAGTCGGC	GCCGACTTCGTGGCGAGCAAGGACGTG
T-Alb-Y334A	GGGTGTCTGCGGGCGGCCTCGTACAGGAACAT	ATGTTCCTGTACGAGGCCGCCCGCAGACACCC
T-Alb-R337A	CTGTAATCGGGGTGTGCGCGGGCGTACTCGTA	TACGAGTACGCCCGCGCACACCCCGATTACAG
T-Alb-H338A	TGTAATCGGGGGCTCTGCGGGCGTACTCGTAC	GTACGAGTACGCCCGCAGAGCCCCCGATTACA
T-Alb-D340A	CACCACGCTGTAAGCGGGGTGTCTGCG	CGCAGACACCCCGCTTACAGCGTGGTG
T-Alb-F374A	AGGCTTGAACTCGTCGGCCACCTTGGCGTAGCAC	GTGCTACGCCAAGGTGGCCGACGAGTTCAAGCCT
T-Alb-V381A	TCTGGGGTTCCTCCGCCAGAGGCTTGAAC	GTTCAAGCCTCTGGCGGAGGAACCCCAGA
T-Alb-E382A	GGTTCTGGGGTTCCGCCACCAGAGGCTTG	CAAGCCTCTGGTGGCGGAACCCCAGAACC
