# Supplementary material for: Direct activation of the proton channel by albumin leads to human sperm capacitation and sustained release of inflammatory mediators by neutrophils
Source: Nat Commun. 2021 Jun 22;12:3855. doi: 10.1038/s41467-021-24145-1 (PMC8219737; doi:10.1038/s41467-021-24145-1)
Supplement: Supplementary file 2 — Reporting Summary [file 41467_2021_24145_MOESM2_ESM.pdf]

nature research

Corresponding author(s): Steve A.N. Goldstein

Last updated by author(s): May 26, 2021

Reporting Summary

Nature Research wishes to improve the reproducibility of the work that we publish. This form provides structure for consistency and transparency in reporting. For further information on Nature Research policies, see our Editorial Policies and the Editorial Policy Checklist.

Statistics

For all statistical analyses, confirm that the following items are present in the figure legend, table legend, main text, or Methods section.

n/a	Confirmed	
☐	☒	The exact sample size (*n*) for each experimental group/condition, given as a discrete number and unit of measurement
☐	☒	A statement on whether measurements were taken from distinct samples or whether the same sample was measured repeatedly
☐	☒	The statistical test(s) used AND whether they are one- or two-sided<br>*Only common tests should be described solely by name; describe more complex techniques in the Methods section.*
☒	☐	A description of all covariates tested
☐	☒	A description of any assumptions or corrections, such as tests of normality and adjustment for multiple comparisons
☒	☐	A full description of the statistical parameters including central tendency (e.g. means) or other basic estimates (e.g. regression coefficient) AND variation (e.g. standard deviation) or associated estimates of uncertainty (e.g. confidence intervals)
☐	☒	For null hypothesis testing, the test statistic (e.g. *F*, *t*, *r*) with confidence intervals, effect sizes, degrees of freedom and *P* value noted<br>*Give P values as exact values whenever suitable.*
☒	☐	For Bayesian analysis, information on the choice of priors and Markov chain Monte Carlo settings
☒	☐	For hierarchical and complex designs, identification of the appropriate level for tests and full reporting of outcomes
☒	☐	Estimates of effect sizes (e.g. Cohen's *d*, Pearson's *r*), indicating how they were calculated

Our web collection on statistics for biologists contains articles on many of the points above.

Software and code

Policy information about availability of computer code

Data collection	pClamp 10, Metamorph 7, SkanIt 2.6, HPEPDOCK web server, NAMD 2.14,
Data analysis	Origin 8, ImageJ windows version, VMD 1.93, Clampfit 10, SigmaPlot 9.0

For manuscripts utilizing custom algorithms or software that are central to the research but not yet described in published literature, software must be made available to editors and reviewers. We strongly encourage code deposition in a community repository (e.g. GitHub). See the Nature Research guidelines for submitting code & software for further information.

Data

Policy information about availability of data

All manuscripts must include a data availability statement. This statement should provide the following information, where applicable:

- Accession codes, unique identifiers, or web links for publicly available datasets
- A list of figures that have associated raw data
- A description of any restrictions on data availability

Data availability
All data needed to evaluate the conclusions in the paper are present in the paper and supplementary information. A reporting summary for this article is available as a Supplementary Information file. Additional data related to this paper are available from the corresponding author upon reasonable request. The source data underlying Figs. 1a-f, 2a-d, 3b, 3d, 4b-d, 5c, 5d, 5g-j, and Supplementary Figs. 1a, 1c, 1f-g, 2b, 3a-c, 5b-c, 9b, 10b, 12b, 19, 20b are provided as a Source Data file.

Code availability
The crystal structure of Alb that was used for molecular dynamics simulation is from Protein Data Bank with accession code 1BM0. The EPR-derived structural model of hHv1 and the computational model of the Alb-hHv1 complex are available from the corresponding author upon reasonable request.

Field-specific reporting

Please select the one below that is the best fit for your research. If you are not sure, read the appropriate sections before making your selection.

☒ Life sciences ☐ Behavioural & social sciences ☐ Ecological, evolutionary & environmental sciences

For a reference copy of the document with all sections, see nature.com/documents/nr-reporting-summary-flat.pdf

Life sciences study design

All studies must disclose on these points even when the disclosure is negative.

Sample size	No sample-size calculation was performed. Sample size in all experiments are ≥ 3. This size was determined based on our previously studies and the standard used in the electrophysiology field. This size is sufficient and it is rationalized by our published papers (reference 5 and 42 in the manuscript).
Data exclusions	No data were excluded.
Replication	All experiments were performed at least three times, and all findings were reproducible.
Randomization	Randomly selected cells/samples were used for recording/measurement.
Blinding	Blinding was not performed. This is because Albumin is the only activator studied in the research and it has unambiguous strong activation effect on hHv1 channel, blinding is not necessary.

Reporting for specific materials, systems and methods

We require information from authors about some types of materials, experimental systems and methods used in many studies. Here, indicate whether each material, system or method listed is relevant to your study. If you are not sure if a list item applies to your research, read the appropriate section before selecting a response.

Materials & experimental systems

n/a	Involved in the study	
☒	☐	Antibodies
☐	☒	Eukaryotic cell lines
☒	☐	Palaeontology and archaeology
☒	☐	Animals and other organisms
☐	☒	Human research participants
☒	☐	Clinical data
☒	☐	Dual use research of concern

Methods

n/a	Involved in the study	
☒	☐	ChIP-seq
☒	☐	Flow cytometry
☒	☐	MRI-based neuroimaging

Eukaryotic cell lines

Policy information about cell lines

Cell line source(s)	HEK293T cells (RRID: CVCL_0063).
Authentication	The cell line used was authenticated by ATCC using morphology, karyotyping, and PCR based approaches.
Mycoplasma contamination	Negative.
Commonly misidentified lines (See ICLAC register)	No commonly misidentified cell lines were used in the study

Human research participants

Policy information about studies involving human research participants

Population characteristics	Ejaculates were obtained from healthy male donors with ages ranging from 22-40 years old by masturbation after at least 48 h of sexual abstinence. Only semen samples that fulfilled the World Health Organization (WHO 2010) guidelines were selected for experiments. The informed consent signed by the donors.<br>Peripheral blood was obtained from healthy donors at Institute for Clinical and Translational Science of University of California Irvine. Donor population is composed of 50% female and 50% male with ages ranging from 23-62 years old.
Recruitment	Participants are recruited at National University of Cuyo, National Autonomous University of Mexico and University of California Irvine. All participants were randomly chosen, no biases.
Ethics oversight	The protocol for semen sample handling were approved by the Ethic Committee of the School of Medicine, National University of Cuyo and the Bioethics Committee at the Biotechnology Institute from the National Autonomous University of Mexico.<br>The blood sample were collected by Institute for Clinical and Translational Science of University of California Irvine and the protocol was approved by the Institutional Review Board of University of California Irvine.

Note that full information on the approval of the study protocol must also be provided in the manuscript.
